# Assembly of a Dihydrideborate and Two Aryl Nitriles to Form a C,N,N′-Pincer Ligand Coordinated to Osmium

**DOI:** 10.1021/acs.organomet.0c00690

**Published:** 2021-03-11

**Authors:** Juan C. Babón, Miguel A. Esteruelas, Israel Fernández, Ana M. López, Enrique Oñate

**Affiliations:** † Departamento de Química Inorgánica, Instituto de Síntesis Química y Catálisis Homogénea (ISQCH), Centro de Innovación en Química Avanzada (ORFEO-CINQA), Universidad de Zaragoza-CSIC, 50009 Zaragoza, Spain; ‡ Departamento de Química Orgánica I, Facultad de Ciencias Químicas, Centro de Innovación en Química Avanzada (ORFEO-CINQA), Universidad Complutense de Madrid, 28040 Madrid, Spain

## Abstract

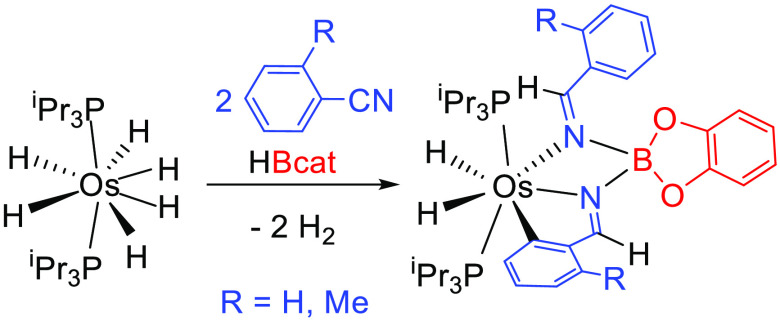

The C,N,N′-donor aryl-diimineborate pincer ligand of the complexes OsH_2_{κ^3^-*C*,*N*,*N*-[C_6_H_3_RCH=NB(cat)N=CHC_6_H_4_R]}(P^i^Pr_3_)_2_ (R = H, Me) has been generated in a one-pot procedure, by the reaction of the hexahydride OsH_6_(P^i^Pr_3_)_2_ with catecholborane (catBH) and two molecules of the corresponding aryl nitrile. The osmium–pincer bonding situation has been analyzed by means of atoms in molecules (AIM), natural bond orbital (NBO), and energy decomposition analysis coupled with the natural orbitals for chemical valence (EDA-NOCV) methods. According to the results, the complexes exhibit a rather strong electron-sharing Os–C bond, two weaker donor–acceptor N–Os bonds, and two π-back-donations from the transition metal to vacant π* orbitals of the formed metallacycles. In addition, spectroscopic findings and DFT calculations reveal that the donor units of the pincer are incorporated in a sequential manner. First, the central Os–N bond is formed, by the reaction of the dihydrideborate ligand of the intermediate OsH_3_{κ^2^-*H*,*H*-(H_2_Bcat)}(P^i^Pr_3_)_2_ with one of the aryl nitriles. The subsequent oxidative addition of the *o*-C–H bond of the aryl substituent of the resulting κ^1^-*N*-(*N*-boryl-arylaldimine) affords the Os–C bond. Finally, the second Os–N bond is generated from a hydride, an ortho-metalated *N*-boryl-arylaldimine, and the second aryl nitrile.

## Introduction

Transition-metal complexes stabilized by pincer ligands are having a great effect on modern chemistry because of their high stability and the broad range of their applications,^1^ which extend from catalysis^2^ and medicine^3^ to materials science.^4^ Thus, platinum-group metal pincer complexes are currently situated at the forefront of important fields such as homogeneous catalysis^5^ and photophysics.^6^ Furthermore, the disposition of their donor atoms at the metal coordination sphere allows them to develop a noticeable ability to form uncommon coordination polyhedra and favor unusual metal oxidation states. As a consequence, rare compounds have been recently discovered, such as the *mer*-tris(boryl) derivative Ir(Bcat)_3_{κ^3^-*P*,*O*,*P*-[xant(P^i^Pr_2_)_2_]} (xant(P^i^Pr_2_)_2_ = 9,9-dimethyl-4,5-bis(diisoproylphosphino)xanthene), which challenges the concept of trans influence,^7^ and families of metallapentalenes, heterometallapentalenes,^8^ and metallapentalynes,^9^ with planar Möbius aromaticity.

Osmapentalenes and osmapentalynes are known.^8,9^ However, pincer complexes of this element are scarce in comparison with the number of known compounds for the rest of the platinum-group metals. The pincer ligands coordinated to this element mainly involve neutral^10^ or monoanionic^10f,11^ moieties, which result from the simple coordination of tridentate molecules^10^ or the coordination of bidentate groups along with the σ-bond activation of one of their substituents,^11^ whereas osmium complexes bearing dianionic pincer ligands are very rare. Examples include C,C′,N-donors, which are generated by coordination of a 2e-donor N atom and two σ-bond activations at the molecule skeleton,^12^ or O,N,O-,^13^ C,C′,C-,^14^ and C,N,C-donors,^15^ which are formed as a consequence of the coordination of a 2e-donor atom and a σ-bond activation at two substituents of the molecular core.^13−15^

This paper describes the discovery of a new class of formally dianionic pincer ligands coordinated to osmium, aryl-diimineborate (C,N,N′), which are furthermore generated on the metal coordination sphere by a novel procedure involving the coupling of a coordinated dihydrideborate anion with two aryl nitrile molecules and the activation of an *o*-C–H bond of the aryl group of one of them.

## Results and Discussion

### Isolation and Characterization of the Complexes

**Scheme 1:**
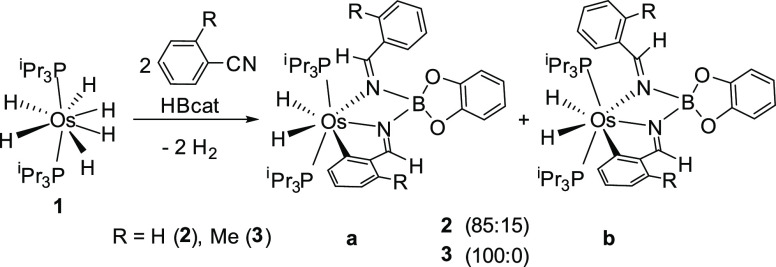
Reactions of Complex **1** with Aryl Nitriles and Catecholborane

The discovery was completely accidental. We were studying the reactions of the hexahydride complex OsH_6_(P^i^Pr_3_)_2_ (**1**) with boranes^16^ and nitriles^17^ as a part of our work on the chemistry of polyhydride complexes of platinum-group metals,^18^ and we observed that while complex **1** promoted the catalytic addition of the B–H bond of catecholborane (catBH) and pinacolborane (pinBH) to alkyl nitriles to give borylimines, which under the reaction conditions evolved to diborylamines,^19^ it was inactive for the same reactions with aryl nitriles. The finding did not surprise us since we had noticed a similar effect of the aryl groups on the reactions of hydrogenation of these substrates to secondary amines. In that case, the intermediate arylaldimine reacted with **1** to give the catalytically unproductive trihydrides OsH_3_{κ^2^-*C*,*N*-(C_6_H_3_RCH=NH)}(P^i^Pr_3_)_2_ (R = H, Me), as a result of the coordination of the nitrogen atom and the activation of an *o*-C–H bond of the aryl substituent.^17b^ To check for the formation of a similar compound resulting from an analogous reaction with a *N*-boryl-arylaldimine, we treated complex **1** with 5 equiv of benzonitrile and 10 equiv of catBH, in toluene at 50 °C for 6 h. To our surprise, the formed species was the dihydride-pincer derivative OsH_2_{κ^3^-*C*,*N*,*N*-[C_6_H_4_CH=NB(cat)N=CHPh]}(P^i^Pr_3_)_2_ (**2**), which was isolated as a 85:15 mixture of the isomers **a** and **b** shown in Scheme 1. Similarly, the reaction with *o*-tolunitrile afforded the methyl-substituted analogue OsH_2_{κ^3^-*C*,*N*,*N*[C_6_H_3_MeCH=NB(cat)N=CHC_6_H_4_Me]}(P^i^Pr_3_)_2_ (**3**), as only one isomer. The methyl group increases the steric hindrance of the aryl substituent of the pincer, and this appears to destabilize isomer **b** with respect to **a**.

**Figure 1:**
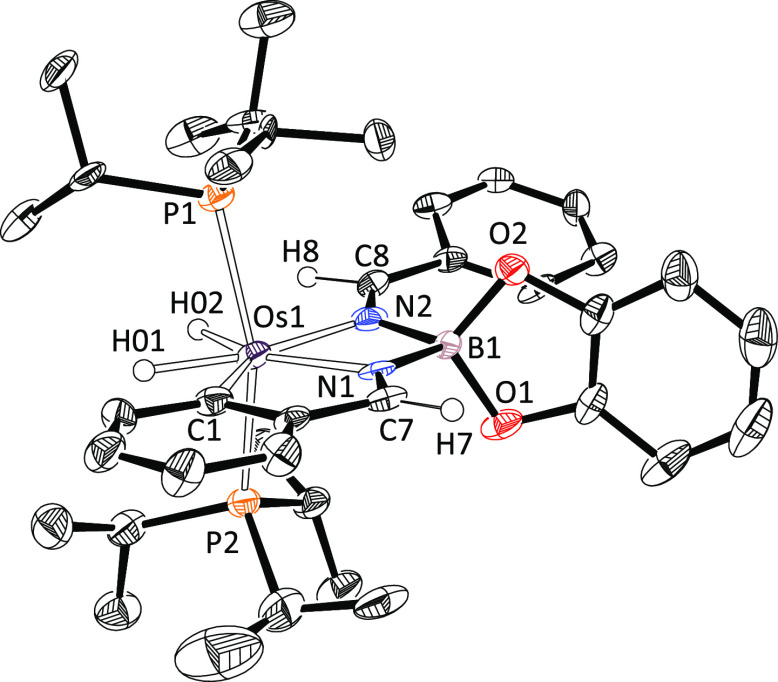
Molecular structure of one of the two independent molecules of complex **2a** (50% probability ellipsoids) in the asymmetric unit. Hydrogen atoms except for hydrides and those attached to C_α_ atoms of the imine groups are omitted for clarity. Selected bond distances (Å) and angles (deg): Os(1)–C(1) = 2.076(12), 2.074(11), Os(1)–N(1) = 2.045(9), 2.021(9), Os(1)–N(2) = 2.137(9), 2.142(10), N(1)–C(7) = 1.263(14), 1.281(14), N(1)–B(1) = 1.556(17), 1.558(14), N(2)–C(8) = 1.282(14), 1.288(14), N(2)–B(1) = 1.561(16), 1.582(15); N(2)–Os(1)–N(1) = 67.2(4), 66.8(3), N(1)–Os(1)–C(1) = 75.2(4), 75.6(4), N(2)–Os(1)–C(1) = 142.3(4), 142.3(4), P(1)–Os(1)–P(2) = 159.48(10), 160.93(10).

Orange crystals of **2a** suitable for X-ray diffraction analysis were obtained at −30 °C from a pentane solution. The structure, which has two molecules that are chemically equivalent but crystallographically independent in the asymmetric unit (Figure 1 shows one of them), proves the formation of the pincer. The tridentate ligand acts with N(2)–Os–N(1), N(1)–Os–C(1), and N(2)–Os–C(1) angles of 67.2(4) and 66.8(3)°, 75.2(4) and 75.6(4)°, and 142.3(4)° in both molecules, respectively. The angle N(2)–Os–C(1) is particularly notable. It is very close to the ideal value of 144° for a pentagonal-bipyramidal arrangement of donor atoms around the metal center, pointing out that these aryl-diimineborate pincer ligands should be especially useful to stabilize complexes of d^4^ ions with a pentagonal-bipyramidal structure,^15a,20^ which is also the observed structure in **2**. The phosphines in the apical positions (P(1)–Os–P(2) = 159.48(10) and 160.93(10)°) and the hydrides, separated by 1.9(1) and 1.7(1) Å, in the pincer plane complete the coordination polyhedron. In agreement with the presence of two inequivalent hydride ligands, the ^1^H NMR spectra of **2** and **3** in benzene-*d*_*6*_ show two doublets (^2^*J*_H–H_ ≈ 21 Hz) of triplets (^2^*J*_H–P_ ≈ 14 Hz) at about −4.5 and −7.5 ppm. In the ^31^P{^1^H} NMR spectra, the equivalent phosphines give rise to a singlet at 6.4 ppm for both compounds. The ^13^C{^1^H} NMR spectra contain a triplet (^2^*J*_C–P_ = 6 Hz) corresponding to the metalated carbon atom of the pincer, between 166 and 164 ppm. A broad signal centered at 14.8 ppm in the ^11^B NMR spectra, due to the catB linker, is also a characteristic of these borates.

**Figure 2:**
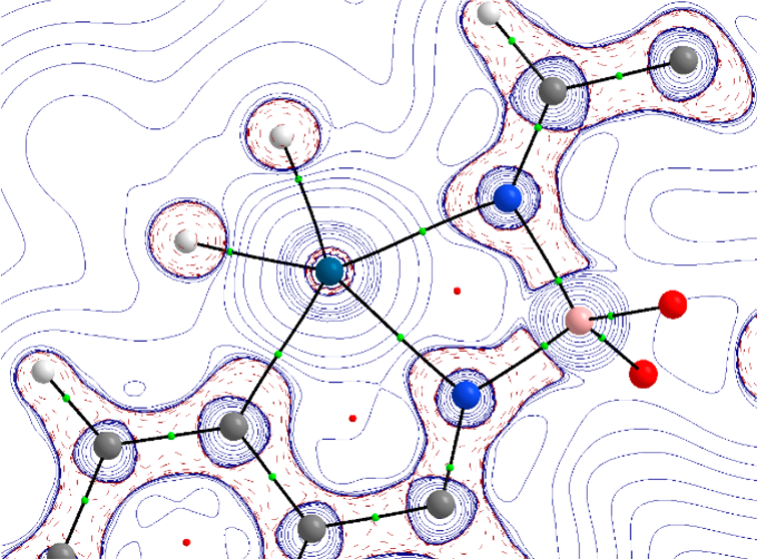
Contour line diagrams ∇^2^ρ(*r*) for complex **2a** in the Os–N–B–N plane. The solid lines connecting the atomic nuclei are the bond paths, while the small green and red spheres indicate the corresponding bond critical points and ring critical points, respectively. Legend: H (white), B (pink), C (gray), Os (dark blue), and N (light blue).

The novelty of this class of pincer ligands prompted us to study their interactions with the metal center. The osmium–pincer bonding situation was analyzed by means of DFT calculations (BP86-D3/def2-SVP) initially using atoms in molecules (AIM) and natural bond orbital (NBO) methods. The AIM method displays the expected Os–C and Os–N bond critical points (BCPs) together with their associated bond paths (BPs) running between these atoms and two additional ring critical points associated with the five (OsNC_3_)- and four-membered (OsN_2_B) rings (Figure 2). Although in all cases the Laplacian distribution in the Os–N–B–N plane exhibits the shape of a droplet directed toward the osmium atom, typical for a donor–acceptor interaction,^21^ the computed delocalization index, δ, which is a measure of the relative bond strength,^22^ indicates that the Os–C bond (δ = 0.89) is significantly stronger than both Os–N bonds (δ = 0.65 and 0.71). A similar result is found by applying the NBO method. The computed Wiberg bond index (WBI) for the Os–C bond (0.67) is markedly higher than the values computed for the Os–N bonds (WBI = 0.40 and 0.42, respectively).

**Figure 3:**
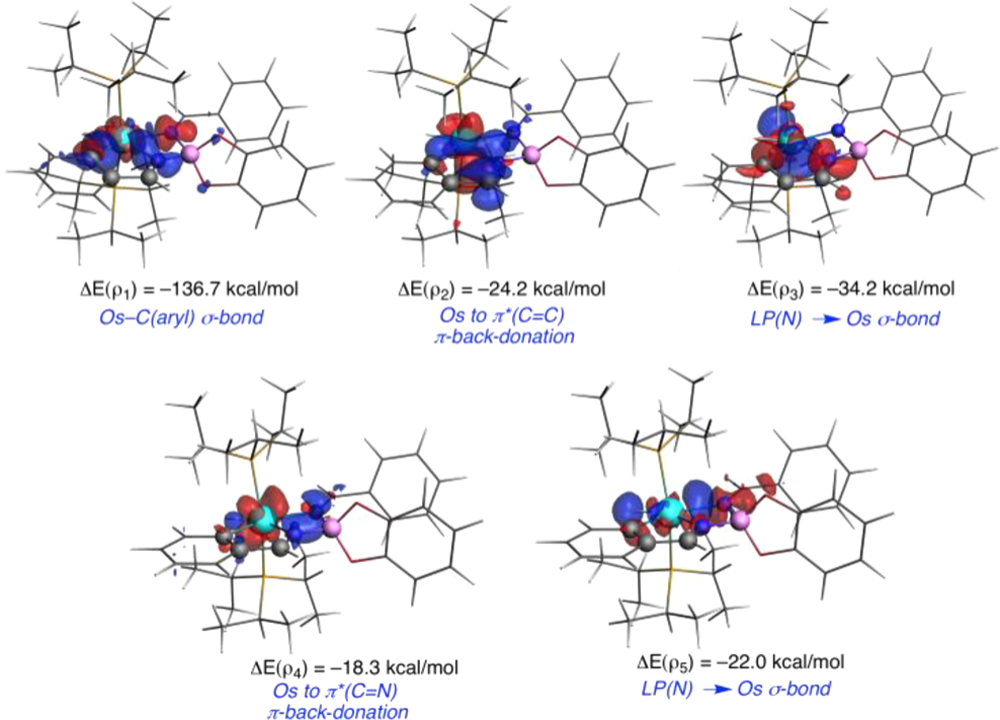
NOCV-deformation densities and associated stabilization energies computed for complex **2a**. The charge flow takes place in the direction red → blue.

The osmium–pincer bonding situation could be described in terms of two donor–acceptor N–Os bonds and an electron-sharing Os–C bond, according to the AIM and NBO results. Figure 3 depicts the main orbital interactions, computed using the energy decomposition analysis coupled with the natural orbitals for chemical valence method (EDA-NOCV). As can be clearly seen, the strengths of both donor–acceptor N–Os bonds are rather similar (−34.2 and −22.0 kcal mol^–1^) and are significantly weaker than the electron-sharing Os–C bond (−136.7 kcal mol^–1^). This agrees with the relative bond strengths suggested by the computed delocalization and Wiberg bond indices. Moreover, the NOCV method identifies two additional π-back-donations from the transition-metal fragment to vacant π*(C=C) and π*(C=N) orbitals of the metallacycles. These π-back-donations, which resemble those found in related fused-ring osmacycles,^23^ have strengths similar to those of the donor–acceptor N–Os bonds (−24.2 and −18.3 kcal mol^–1^, respectively). Therefore, the metal–pincer interaction in **2a** exhibits a rather strong electron-sharing Os–C bond, two weaker donor–acceptor N–Os bonds and two π-back-donations from the transition-metal fragment to vacant π* orbitals of the formed metallacycles.

### Mechanism of the Formation of the Pincer

**Figure 4:**
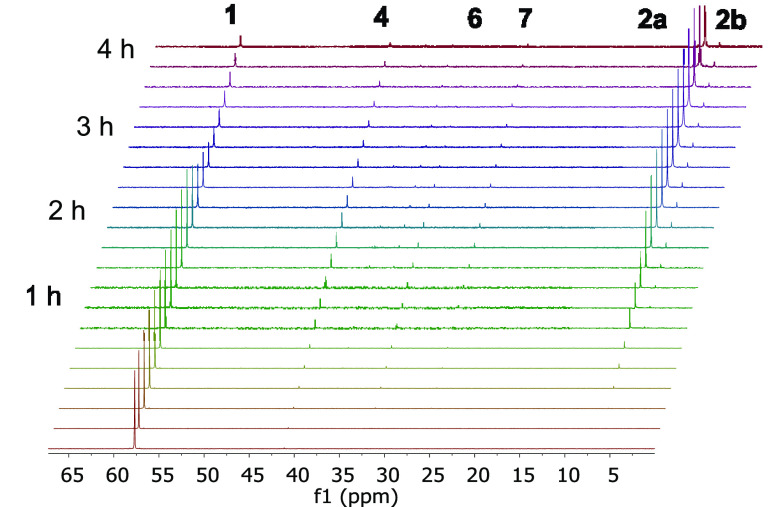
^31^P{^1^H} NMR spectra of the reaction of **1** (0.1 M) with benzonitrile (0.5 M) and catecholborane (1.0 M) (1:5:10 molar ratio) in toluene-*d*_8_ at 50 °C as a function of time. Spectra were recorded at regular intervals of 12 min over 4 h.

Complex **1** individually reacts with both catBH and benzonitrile. The reaction with catBH affords the trihydride dihydrideborate OsH_3_{κ^2^-*H*,*H*-(H_2_Bcat)}(P^i^Pr_3_)_2_ (**4**),^16^ whereas the reaction with benzonitrile gives the trihydride azavinylidene OsH_3_(=N=CHPh)(P^i^Pr_3_)_2_ (**5**).^17b^ Thus, in principle, either of the compounds could be the precursor of **2**. In the search for understanding the formation of the pincer ligand, we followed the reaction of **1** with HBcat and benzonitrile by ^31^P{^1^H} NMR spectroscopy, as a function of time (Figure 4). The spectra show the initial formation of the trihydride dihydrideborate **4**, which is transformed into an isomeric mixture of **2a** and **2b**, via the two unknown species **6** and **7**. No traces of the azavinylidene **5** were detected. This is consistent with previous DFT calculations suggesting that the activation barrier for the formation of **4** is significantly lower than that for the formation of **5**.^17b,19^ To confirm the participation of complex **4** in the formation of **2**, we added 1.0 equiv of benzonitrile to a toluene solution of this complex contained in a NMR tube, at −78 °C. As expected, the quantitative formation of **6** took place. It decomposes to an ill-defined mixture, at room temperature. However, in the presence of 4 equiv of benzonitrile, complex **6** evolves into **2**, via **7** detected in the ^31^P{^1^H} NMR spectra (δ_^31^P_ 26.0).

The ^11^B, ^1^H, and ^13^C{^1^H} spectra of **6** strongly support the presence of an imine-hydrideborate anion coordinated to the metal center. Thus, the first spectrum contains a broad signal centered at −6.7 ppm; the second spectrum shows a downfield imine resonance at 9.08 ppm, coupled in ^1^H–^13^C HSQC to a ^13^C NMR signal at 156.3 ppm, along with an HB resonance at −4.97 ppm. The ^1^H NMR spectrum at 183 K also reveals the presence of three inequivalent hydrides in the coordination plane of the borate, with three signals at −8.86, −10.62, and −10.80 ppm, whereas the ^31^P{^1^H} NMR spectrum displays a singlet at 30.5 ppm consistent with equivalent phosphine ligands. According to these spectroscopic features, intermediate **6** is the osmium(IV) species OsH_3_{κ^2^-*H*,*N*-[HB(cat)N=CHPh]}(P^i^Pr_3_)_2_, resulting from the insertion of the N–C triple bond of the nitrile in one of the B–H bonds of the dihydrideborate of **4**.

Complexes bearing imine-hydrideborate ligands are known but are scarce. Milstein and co-workers have reported that the reaction of FeBr_2_ with 4,5-bis(diphenylphosphino)acridine (^HACR^PNP) in an acetonitrile solution containing 2 equiv of NaBH_4_ gives Fe{κ^2^-*H*,*N*-[H-BH_2_–N=CHMe]}(MeC≡N){κ^3^*-P*,*N*,*P*-(^HACR^PNP)},^24^ whereas Berke and co-workers have prepared the rhenium complex Re{κ^2^-*H*,*N*-[H-BH_2_–N=CHMe]}Cl(PMe_3_)_2_(NO) by addition of [BH_4_]^−^ to the acetonitrile precursor ReCl_2_(MeC≡N)(PMe_3_)_2_(NO).^25^ The Nikonov group has observed that the addition of catBH to the azavinylidene-molybdenum compound Mo(=N-2,6-^i^Pr_2_C_6_H_3_)(=N=CHPh)Cl(PMe_3_)_2_ affords Mo{κ^2^-*H*,*N*-[HB(cat)N=CHPh]}(=N-2,6-^i^Pr_2_C_6_H_3_)Cl(PMe_3_)_2_, bearing the same imine-hydrideborate as **6**.^26^

Intermediate **7** is most probably the ortho-metalated borylaldimine derivative OsH_3_{κ^2^-*N*,*C*-[N(Bcat)=CHC_6_H_4_]}(P^i^Pr_3_)_2_, according to the chemical shift of its associated signal in the ^31^P{^1^H} NMR spectra of Figure 4, which resembles that reported for the pinacolborane counterpart OsH_3_{κ^2^-*N*,*C*-[N(Bpin)=CHC_6_H_4_]}(P^i^Pr_3_)_2_ (δ_^31^P_ 26.0).^17b^ Its formation should be the result of the release of a hydrogen molecule from **6** and the *o*-C–H bond activation of the phenyl substituent of the imine moiety, in agreement with the marked ability of the osmium-polyhydrides to activate C–H bonds.^18^

**Figure 5:**
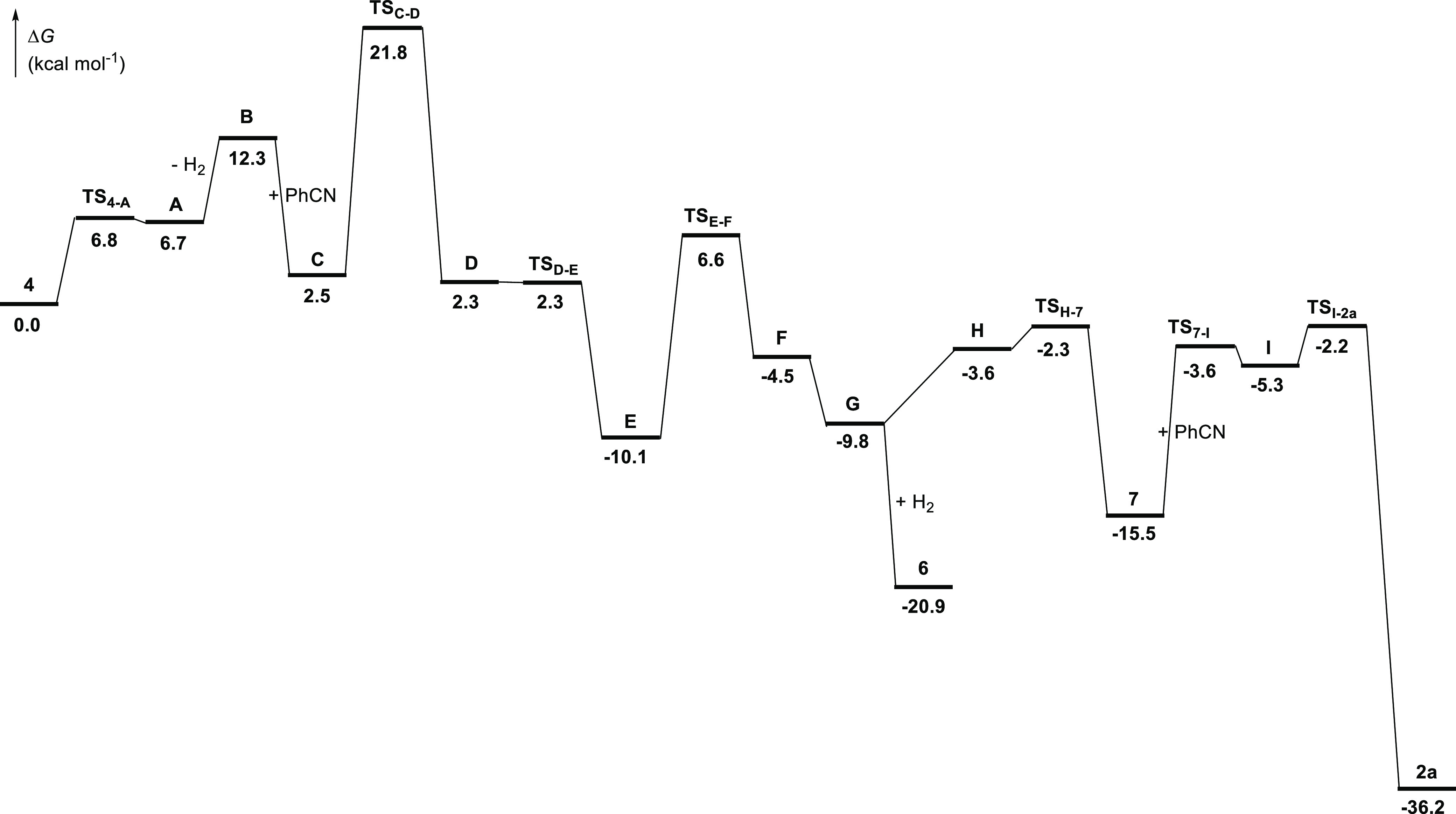
DFT-computed energy profile for the formation of complex **2a**.

**Scheme 2:**
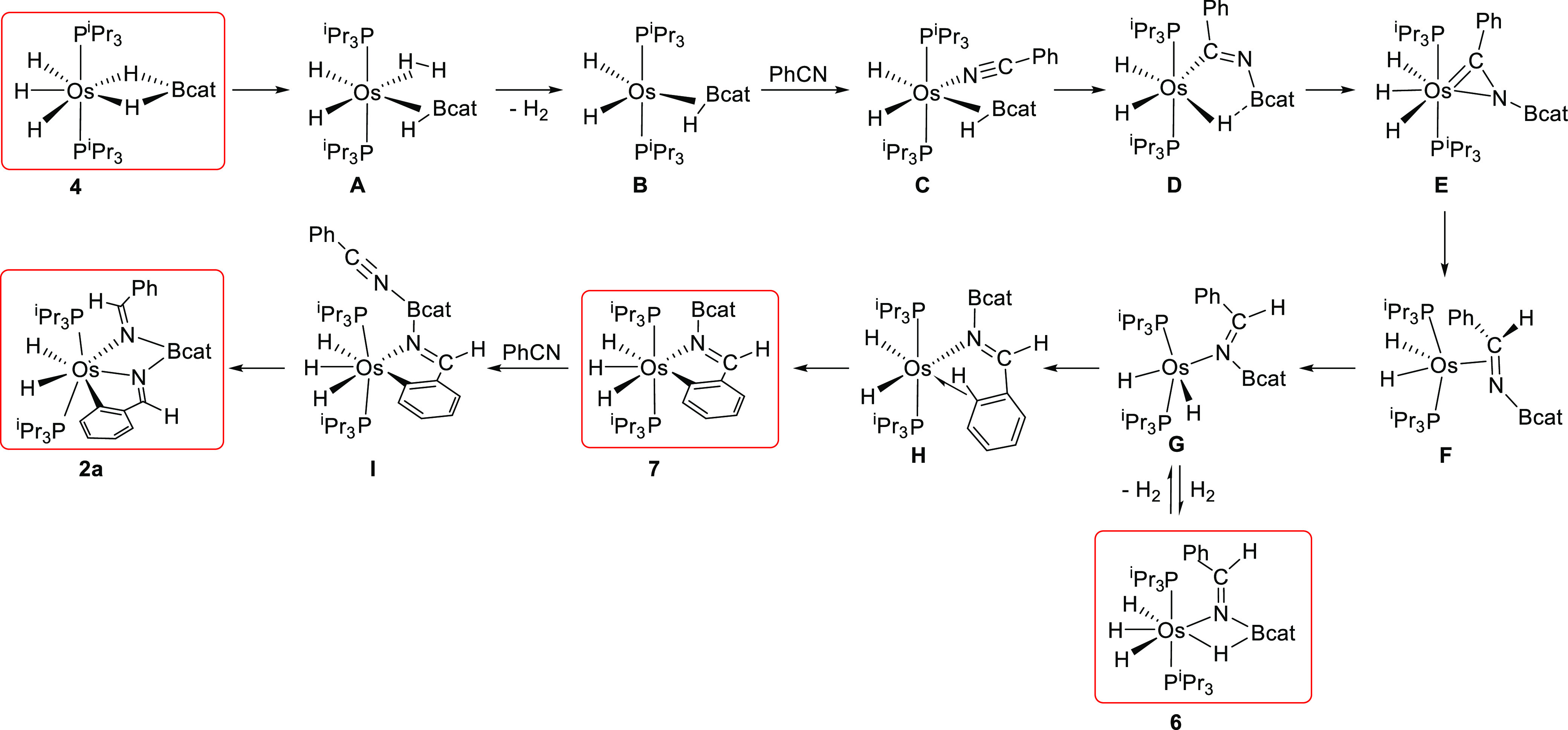
Intermediates for the Formation of Complex **2a** Isolated or detected species are shown in red boxes.

Once the sequence of key intermediates for the arrangement of the pincer had been experimentally established, we decided to analyze the formation of **6** and **7** starting from **4** and the transformation of **7** into **2**. To gain information on the intimate details of the processes, we carried out DFT calculations at the dispersion-corrected PCM(toluene)-B3LYP-D3/def2-SVP level (see computational details in the Supporting Information). The changes in free energy (Δ*G*) were calculated in toluene at 298.15 K and 1 atm. Figure 5 shows the computed energy profile, which displays activation energies lower than 22.0 kcal mol^–1^ with respect to the origin, whereas Scheme 2 gathers all of the intermediates involved in the reaction.

The formation of the pincer can be split into three stages, each of them involving the elemental steps for the formation of the respective donor units. The first stage corresponds to the formation of the central Os–N bond and its elemental steps allow rationalizing the isolation of **6**, which is not a productive intermediate in the formation of the pincer but a side species. During the second stage the Os–C bond is generated and ends up in **7**. The third stage involves the steps leading to the other Os–N bond and shows the transformation of **7** into the pincer complex.

The first stage begins with the activation of the precursor **4**. This trihydride dihydrideborate complex is saturated, and therefore its reaction with the nitrile requires the previous creation of a coordination vacancy. This occurs by dissociation of a hydrogen molecule through the Kubas type dihydrogen (*d*_H–H_ = 0.900 Å)^18^ σ-borane (*d*_B–H_ = 1.339 Å)^16^ intermediate **A**. The dissociation leads to **B**, producing a destabilization of 12.3 kcal mol^–1^ with respect to **4**. As expected, the subsequent coordination of the nitrile is an exergonic process, which produces a stabilization of 9.8 kcal mol^–1^. Thus, the resulting six-coordinate intermediate **C** is only 2.5 kcal mol^–1^ less stable than **4**. It evolves by means of the insertion of the C–N triple bond of the nitrile into the Os–B bond of the Os σ-borane. The insertion, which is consistent with the nucleophilicity of the nitrogen atom and the electrophilicity of the carbon atom of the nitrile,^27^ has to overcome an activation energy of 19.3 kcal mol^–1^. The boryl migration initially affords **D**, which evolves into the trihydride osmium(IV) *N*-boryl-osmaazacyclopropene intermediate **E**, by rupture of the boryl–hydride interaction. Intermediate **E** is 10.1 kcal mol^–1^ more stable than **4** minus H_2_ plus benzonitrile. The following step is a 1,2-hydride displacement from the metal center to the carbon atom of the three-membered heterometallaring. The barrier for this shift (16.7 kcal mol^–1^) is 2.6 kcal mol^–1^ lower than that computed for the migration of the boryl group to the nitrogen atom of the nitrile. Because the process leads to the η^2^-borylimine intermediate **F**, with the metal center in oxidation state +2, it can be viewed as a reductive elimination reaction. Once **F** is formed, the metal center undergoes a slippage from the coordinated C–N double bond to the nitrogen atom to afford the κ^1^-N isomer **G**. The latter is only 0.3 kcal mol^–1^ less stable than the osmium(IV) precursor **E**. The oxidative addition of the hydrogen molecule, released in the activation process of **4**, to the metal center of **G** directly yields **6**, which is 20.9 kcal mol^–1^ more stable than **4** plus benzonitrile.

The unsaturated metal center of intermediate **G** can alternatively undergo the oxidative addition of an *o*-C–H bond of the phenyl substituent of the borylimine. The reaction takes place via the Os-(σ-CH) intermediate **H**, which lies 6.2 kcal mol^–1^ above **G**. It leads to **7**, which is 5.4 kcal mol^–1^ less stable than **6**. The higher stability of **6** with regard to **7** explains why it is possible to isolate **6** at −78 °C, when **4** reacts with 1.0 equiv of nitrile, while the small difference in stability between them is consistent with the observation of both, at 50 °C, during the course of the pincer formation. Complex **7** is a true key intermediate in the formation of the pincer, in contrast to **6**. This is because its boron atom displays a significant increase in electrophilicity with respect to that of the boron atom of catBH and **6**. As a proof of concept of this finding, it should be pointed that the computed positive charge on this atom in the sequence catBH, **6**, and **7** increases in the following manner: +0.96 < +1.07 < +1.34. As a consequence of this increased electrophilicity, the boron atom of **7** is able to undergo the nucleophilic attack of a second benzonitrile molecule. The resulting adduct **I** allows one of the hydride ligands to approach the C(sp) atom of the nitrile, which permits the Os to C 1,2-hydride shift, with a low barrier of 13.3 kcal mol^–1^, and therefore the formation of the pincer of **2a** in an overall exergonic process by 36.2 kcal mol^–1^.

The presence of a small amount of **2b** in the reaction mixtures shown in Figure 4 suggests an accessible activation barrier, under the reaction conditions, for the rotation of the CHPh moiety of the pincer ligand around the exocyclic C–N double bond.

## Concluding Remarks

This study shows the discovery of a new class of pincer ligands, C,N,N′-donor aryl-diimineborate, which is particularly appropriate to stabilize pentagonal-bipyramidal structures. The osmium–pincer bonding situation includes a rather strong electron-sharing Os–C bond, two weaker donor–acceptor N–Os bonds and two π-back-donations from the metal fragment to vacant π* orbitals of the formed metallacycles. The pincer ligands have been generated on the metal coordination sphere of an osmium(IV) center from a dihydrideborate ligand and two aryl nitriles. Spectroscopic findings and DFT calculations have revealed that the formation of the donor units of the pincer is a sequential process, which takes place in three stages. The first involves the formation of the central Os–N bond, by the reaction of the dihydrideborate ligand and an external aryl nitrile. During the second stage, the Os–C bond is generated by oxidative addition of the *o*-C–H bond of the aryl substituent of a κ^1^-*N*-(*N*-boryl-arylaldimine) ligand. The third stage, which is initiated by the nucleophilic attack of a second aryl nitrile to the boron atom of the resulting ortho-metalated N-boryl-arylaldimine, gives rise to the Os–N′ bond.

In summary, a novel class of pincer ligands has been generated on the coordination sphere of an osmium(IV) metal center, in a one-pot procedure, by the assembly of catecholborane and two aryl nitriles and the corresponding formation mechanism has been established on the basis of spectroscopic observations and DFT calculations.

## Experimental Section

All manipulations were performed with rigorous exclusion of air at an argon/vacuum manifold using standard Schlenk-tube or glovebox techniques. Solvents were dried by the usual procedures and distilled under argon prior to use or obtained oxygen- and water-free from an MBraun solvent purification apparatus. Pentane was stored over P_2_O_5_ in the glovebox. Toluene was stored over sodium in the glovebox. The complex OsH_6_(P^i^Pr_3_)_2_ (**1**)^28^ was prepared according to the published methods. Instrumentation for characterization, X-ray information, and computational details are given in the Supporting Information. Chemical shifts (in parts per million) in the NMR spectra are referenced to residual solvent peaks (^1^H, ^13^C{^1^H}), external H_3_PO_4_ (^31^P{^1^H}), or BF_3_·OEt_2_ (^11^B{^1^H}). Coupling constants, *J*, and *N* (*N* = ^3^*J*_H–P_ + ^5^*J*_H–P′_ for ^1^H or ^1^*J*_C–P_ + ^3^*J*_C–P′_ for ^13^C) are given in hertz.

### Preparation of OsH_2_{κ^3^-*C*,*N*,*N*-[C_6_H_4_CH=NB(cat)N=CHPh]}(P^i^Pr_3_)_2_ (**2**)

Benzonitrile (103 μL, 1.0 mmol) and catecholborane (214 μL, 2.0 mmol) were added to a solution of **1** (100 mg, 0.2 mmol) in 2 mL of toluene. The resulting solution was heated at 50 °C for 6 h. The crude reaction mixture was concentrated to dryness under reduced pressure, giving an orange oil. The addition of pentane at −78 °C afforded an orange solid that was washed with pentane (2 × 1 mL) and dried *in vacuo*. Yield: 63 mg (38%) The low isolated yield is due to the high solubility of complex **2** in the usual organic solvents. NMR data showed the presence of two isomers in an 85:15 molar ratio. Orange single crystals of the major isomer **2a** suitable for X-ray diffraction analysis were grown from a solution of **2** in pentane at −30 °C. NMR. Anal. Calcd for C_38_H_59_BN_2_O_2_OsP_2_: C, 54.41; H, 7.09; N, 3.34. Found: C, 54.69; H, 6.94; N, 3.22. HR-MS (electrospray): *m*/*z* calcd for C_38_H_59_BN_2_O_2_OsP_2_ [M]^+^ 840.3754; found 840.3731. ^1^H NMR (300.13 MHz, C_6_D_6_, 298 K): δ 9.40 (br, 1H, NCH), 8.98 (br, 1H, NCH), 8.13 (d, ^3^*J*_H–H_ = 7.8, 2H, CH Ph), 8.04 (d, ^3^*J*_H–H_ = 7.6, 1H, CH Ph), 7.46 (m, 1H, CH Ph), 7.32 (m, 2H, Bcat), 7.20 (m, 2H, CH Ph), 7.12 (m, 2H, CH Ph), 7.05 (m, 2H, Bcat), 6.97 (m, 1H, CH Ph), 2.10 (m, 6H, CH ^i^Pr), 1.13 (dvt, ^3^*J*_H–H_ = 6.8, *N* = 12.7, 18H, CH_3_^i^Pr), 1.04 (dvt, ^3^*J*_H–H_ = 6.9, *N* = 12.9, 18H, CH_3_^i^Pr), −4.44 (dt, ^2^*J*_H–H_ = 20.8, ^2^*J*_H–P_ = 13.1, 1H, OsH), −7.55 (dt, ^2^*J*_H–H_ = 20.8, ^2^*J*_H–P_ = 14.4, 1H, OsH). ^31^P{^1^H} NMR (121.4 MHz, C_7_D_8_, 298 K): δ 6.4 (s). ^11^B NMR (128.38 MHz, C_7_D_8_, 298 K): δ 14.7 (br). ^13^C{^1^H} APT NMR (75.48 MHz, C_7_D_8_, 298 K): δ 171.4 (s, CH Ph), 164.0 (t, ^2^*J*_C–P_ = 6.0, Os–C), 163.4, 162.0 (both s, NCH), 153.6, 152.8 (both s, C_q_ Ph), 145.3, 144.7 (both CH Ph), 136.6 (C_q_ Bcat), 130.3, 129.2, 127.7, 120.5 (all CH Ph), 119.0, 109.4 (both s, CH Bcat), 26.1 (vt, *N* = 24.4, CH ^i^Pr), 19.9, 19.7 (both s, CH_3_^i^Pr).

### Preparation of OsH_2_{κ^3^-*C*,*N*,*N*-[C_6_H_3_MeCH=NB(cat)N=CHC_6_H_4_Me]}(P^i^Pr_3_)_2_ (**3**)

*o-*Tolunitrile (30 μL, 0.25 mmol) and catecholborane (53.5 μL, 0.5 mmol) were added to a solution of **1** (25 mg, 0.05 mmol) in 2 mL of toluene. The resulting solution was heated at 50 °C for 18 h. The crude reaction mixture was concentrated to dryness under reduced pressure, giving an orange oil. A concentrated solution of the oil in pentane was kept at −30 °C for 18 h to give an orange solid, which was dried in vacuo. Yield: 18 mg (40%). The low isolated yield is due to the high solubility of complex **3** in the usual organic solvents. Anal. Calcd for C_40_H_63_BN_2_O_2_OsP_2_: C, 55.42; H, 7.33; N, 3.23. Found: C, 55.03; H, 7.07; N, 3.08. ^1^H NMR (300.13 MHz, C_6_D_6_, 298 K): δ 9.69 (br, 1H, NCH), 9.57 (br, 1H, NCH), 9.28 (d, ^3^*J*_H–H_ = 7.7, 1H, CH C_6_H_3_Me), 7.87 (d, ^3^*J*_H–H_ = 7.7, 1H, C_6_H_4_Me), 7.34 (m, 1H, C_6_H_3_Me), 7.29 (m, 2H, Bcat), 7.13 (m, 2H, CH C_6_H_4_Me + C_6_H_3_Me), 7.02 (m, 2H, Bcat), 6.92 (m, 1H, C_6_H_4_Me), 6.76 (m, 1H, C_6_H_4_Me), 2.34 (s, 3H, Me), 2.15 (m, 6H, CH ^i^Pr), 2.08 (s, 3H, Me), 1.17 (dvt, ^3^*J*_H–H_ = 6.8, *N* = 12.4, 18H, CH_3_^i^Pr), 1.04 (dvt, ^3^*J*_H–H_ = 7.2, *N* = 12.8, 18H, CH_3_^i^Pr), −4.29 (dt, ^2^*J*_H–H_ = 22.5, ^2^*J*_H–P_ = 14.5, 1H, OsH), −7.55 (dt, ^2^*J*_H–H_ = 22.5, ^2^*J*_H–P_ = 14.7, 1H, OsH). ^31^P{^1^H} NMR (121.4 MHz, C_6_D_6_, 298 K): δ 6.3 (s). ^11^B NMR (128.38 MHz, C_6_D_6_, 298 K): δ 14.9 (br). ^13^C{^1^H} APT NMR (75.48 MHz, C_6_D_6_, 298 K): δ 165 (t, ^2^*J*_C–P_ = 6.0, Os–C), 160.0, 159.7 (both s, NCH), 153.5 (s, C_q_ Ar), 142.5 (s, CH Ar), 139.9, 137.2 (both s, C_q_ Ar), 134.7 (s, C_q_ Bcat), 130.7, 130.0, 129.0, 127.1, 124.7, 122.0 (all s, CH Ar), 119.0, 109.4 (both s, CH Bcat), 25.8 (vt, *N* = 24.6, CH ^i^Pr), 20.0, 19.6 (both s, CH_3_^i^Pr), 18.8, 18.3 (both s, Me).

### Reaction of OsH_3_{κ^2^-*H*,*H*-(H_2_Bcat)}(P^i^Pr_3_)_2_ (**4**) with Benzonitrile: Formation of OsH_3_{κ^2^-*H*,*N*-[HB(cat)N=CHPh)]}(P^*i*^Pr_3_)_2_ (**6**)

Catecholborane (5.4 μL, 0.05 mmol) was placed in an NMR tube containing a solution of **1** (25 mg, 0.05 mmol) in 0.5 mL of toluene-*d*_8_. The tube was heated for 18 h at 50 °C, giving a mixture of **4** and OsH(η^3^-H_2_Bcat)(η^2^-HBcat)(P^i^Pr_3_)_2_ in a 90:10 molar ratio.^16^ After this time, the NMR tube was cooled to −78 °C and benzonitrile (25.8 μL, 0.25 mmol) was added, giving the immediate and quantitative transformation of **4** into **6**. ^1^H NMR (400.13 MHz, C_7_D_8_, 243 K): δ 9.08 (s, 1H, NCH), 8.37 (m, 2H, *o*-CH Ph), 7.26 (m, 2H, *m*-CH Ph), 7.15 (m, 2H, Bcat), 7.22 (m, 1H, *p*-CH Ph), 6.96 (m, 2H, Bcat), 2.23 (m, 6H, CH ^i^Pr), 1.28 (dvt, ^3^*J*_H–H_ = 6.8, *N* = 13.4, 18H, CH_3_^i^Pr), 1.09 (dvt, ^3^*J*_H–H_ = 6.6, *N* = 12.6, 18H, CH_3_^i^Pr), −5.06 (br, 1H, OsHB), −9.88 (br, 2H, OsH_2_), −10.79 (br, 1H, OsH). ^1^H NMR (400.13 MHz, C_7_D_8_, 183 K): δ 9.89 (s, 1H, *o*-CH Ph), 9.09 (s, 1H, 1H, NCH), 7.41 (br, 2H, *m*-CH Ph), 7.25 (m, 2H, Bcat), 7.13 (br, 1H, *p*-CH Ph), 6.95 (m, 2H, Bcat), 6.73 (s, 1H, *o*-CH Ph), 2.16 (br, 6H, CH ^i^Pr), 1.29 (br, 18H, CH_3_^i^Pr), 1.01 (br, 18H, CH_3_^i^Pr), −4.97 (br, 1H, OsHB), −8.86, −10.62, −10.80 (all br, 1H each, OsH_3_). ^31^P{^1^H} NMR (121.4 MHz, C_7_D_8_, 243 K): δ 30.5 (s). ^11^B NMR (128.38 MHz, C_7_D_8_, 243 K): δ −6.7 (br). ^13^C{^1^H} APT NMR (75.48 MHz, C_7_D_8_, 243 K): δ 156.3 (s, NCH), 151.9 (s, C_q_ Bcat), 137.4 (s, C_q_ Ph), 130.1 (s, *o*-CH Ph), 129.0 (s, *p*-CH Ph), 128.1 (s, *m*-CH Ph), 119.8, 110.2 (both s, CH Bcat), 27.1 (vt, *N* = 23.8, CH ^i^Pr), 20.5, 19.7 (both s, CH_3_^i^Pr).

### Monitoring of the Reaction of **1** with Benzonitrile and Catecholborane by ^31^P{^1^H} NMR Spectroscopy: Formation of OsH_3_{κ^2^-*H*,*H*-(H_2_Bcat)(P^i^Pr_3_)_2_ (**4**), OsH_3_{κ^2^-*H*,*N*-[N(=CHPh)Bcat(H)]}(P^*i*^Pr_3_)_2_ (**6**), OsH_3_{κ^2^-*N*,*C*-[N(Bcat)=CHC_6_H_4_]}(P^i^Pr_3_)_2_ (**7**), and OsH_2_{κ^3^-*C*,*N*,*N*-[C_6_H_4_CH=NB(cat)N=CHPh]}(P^i^Pr_3_)_2_ (**2**)

An NMR tube was charged with catecholborane (53.5 μL, 0.5 mmol), benzonitrile (25.8 μL, 0.25 mmol), **1** (25 mg, 0.05 mmol), and 0.5 mL of toluene-*d*_8_. The mixture was heated at 50 °C for 4 h, and the reaction was monitored by ^31^P{^1^H} NMR. The monitoring of these reactions by ^31^P{^1^H} NMR (Figure 4) showed the formation of complexes **2**, **4**, **6**, and **7**.

## Supporting Information Available

General information, NMR spectra, structural analysis, and computational details (PDF)
Cartesian coordinates of all molecules, intermediates, and transition states (XYZ)

## Supplementary Material

om0c00690_si_001.pdf

om0c00690_si_002.xyz
