# Supplementary material for: Assembly of a Dihydrideborate and Two Aryl Nitriles to Form a C,N,N′-Pincer Ligand Coordinated to Osmium
Source: Organometallics. 2021 Mar 11;40(6):635–42. doi: 10.1021/acs.organomet.0c00690 (PMC9180356; doi:10.1021/acs.organomet.0c00690)
Supplement: Supplementary file 1 — om0c00690_si_001.pdf [file om0c00690_si_001.pdf]

Supporting Information

Assembly of a Dihydrideborate and two Aryl Nitriles to Form a C,N,N'-Pincer Ligand Coordinated to Osmium

Juan C. Babón,[†] Miguel A. Esteruelas,[*,†] Israel Fernández,[‡] Ana M. López,[†] and Enrique Oñate[†]

[†]Departamento de Química Inorgánica, Instituto de Síntesis Química y Catálisis Homogénea (ISQCH), Centro de Innovación en Química Avanzada (ORFEO-CINQA), Universidad de Zaragoza-CSIC, 50009 Zaragoza, Spain.

[‡]Departamento de Química Orgánica I, Facultad de Ciencias Químicas, Centro de Innovación en Química Avanzada (ORFEO-CINQA), Universidad Complutense de Madrid, 28040 Madrid, Spain.

* Corresponding author's e-mail address: maester@unizar.es

Contents:

Experimental Section: General Information…………………………………………..….. S2

NMR Spectra ……………………..……………………………………………… S2

Structural Analysis of complex **2a**……………………………………………...…….. S9

Computational Details and Energies of Computed Structures……………………… S10

References…..…………………………………………………………….………… S17

Experimental Section: General Information

NMR spectra were recorded on a Bruker ARX 300, a Bruker Avance 300 MHz, or a Bruker Avance 400 MHz. C, H, and N analyses were carried out in a LECO CHNS-932 analyzer. High-resolution (HRMS) electrospray mass spectra were acquired using a MicroTOF-Q hybrid quadrupole time-of-flight spectrometer.

NMR Spectra

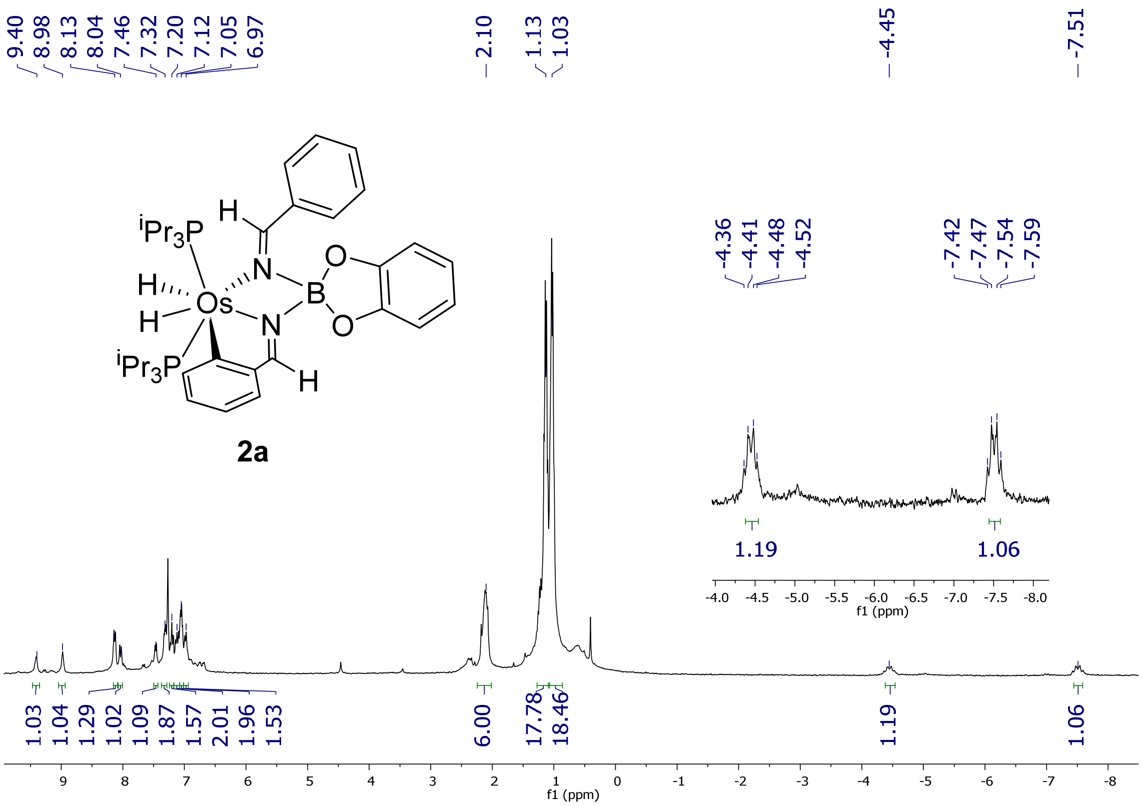

Figure S1. ^{1}H NMR (300.13 MHz, C_6D_6, 298 K) spectrum for complex **2a**.

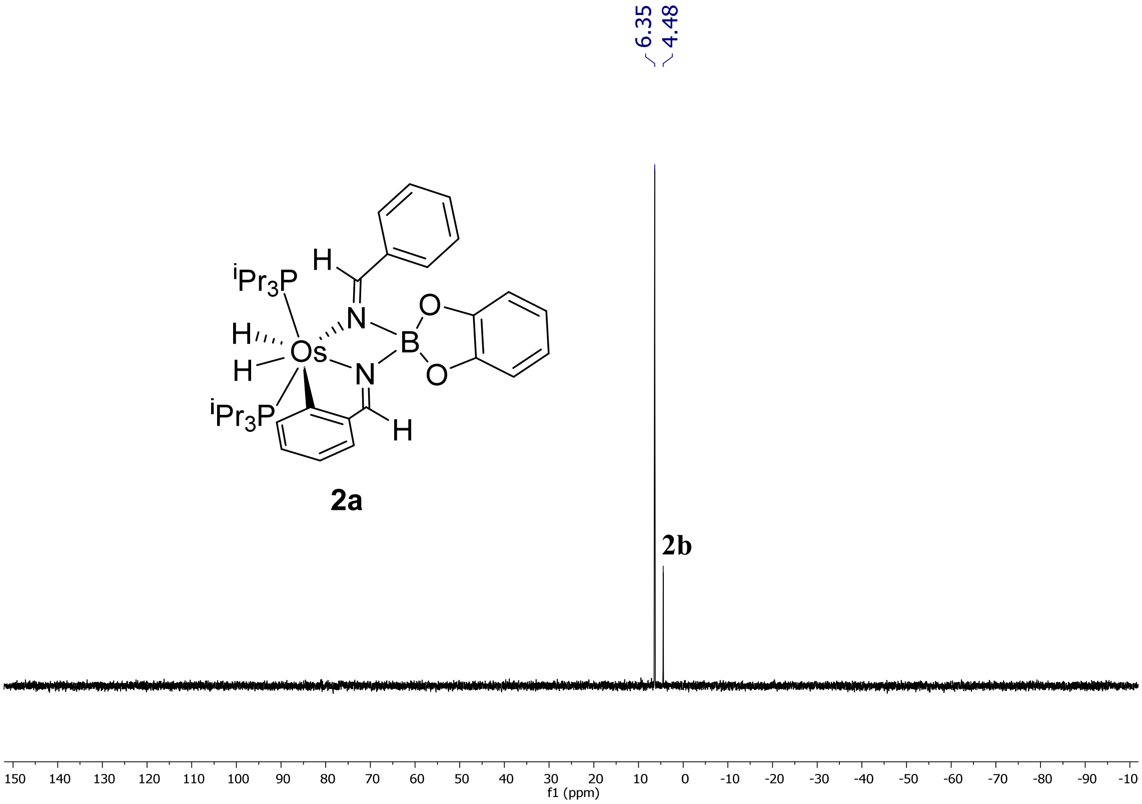

Figure S2. $^{31}P\{^{1}H\}$ NMR (121.50 MHz, C_6D_6, 298 K) spectrum for complex **2a**.

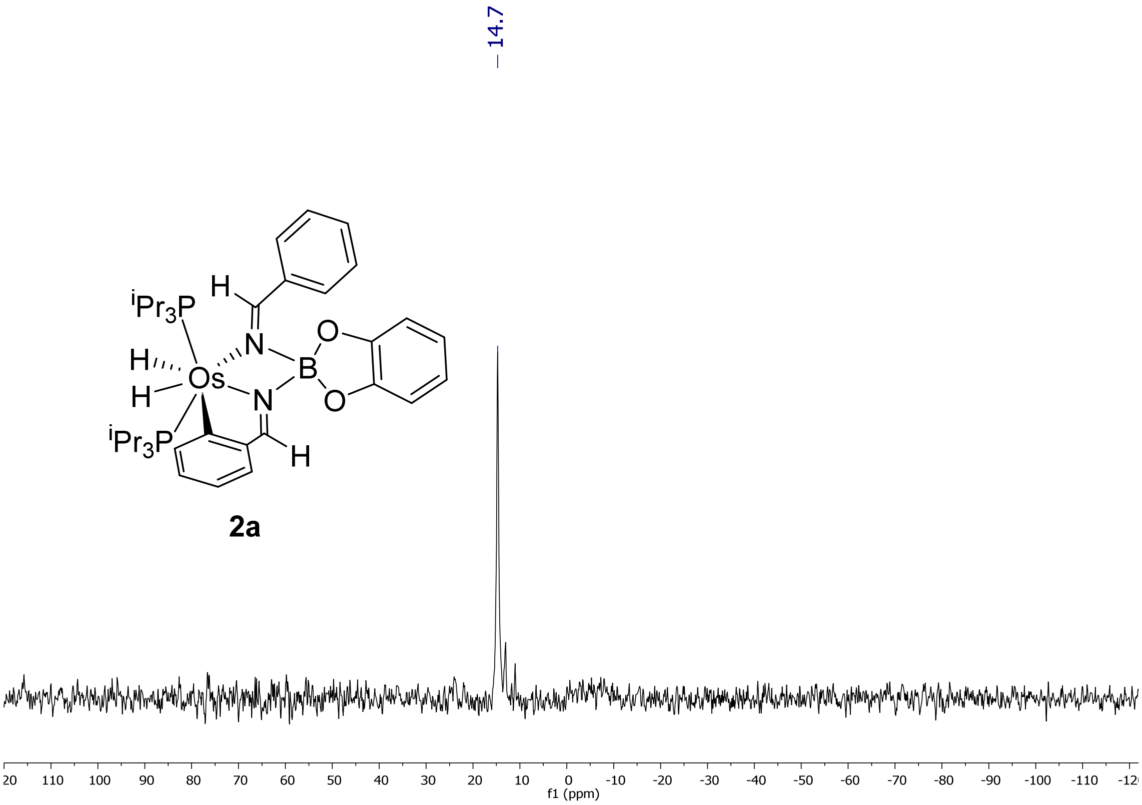

Figure S3. $^{11}B\{^{1}H\}$ APT NMR (96.29 MHz, C_6D_6, 298 K) spectrum of complex **2a**.

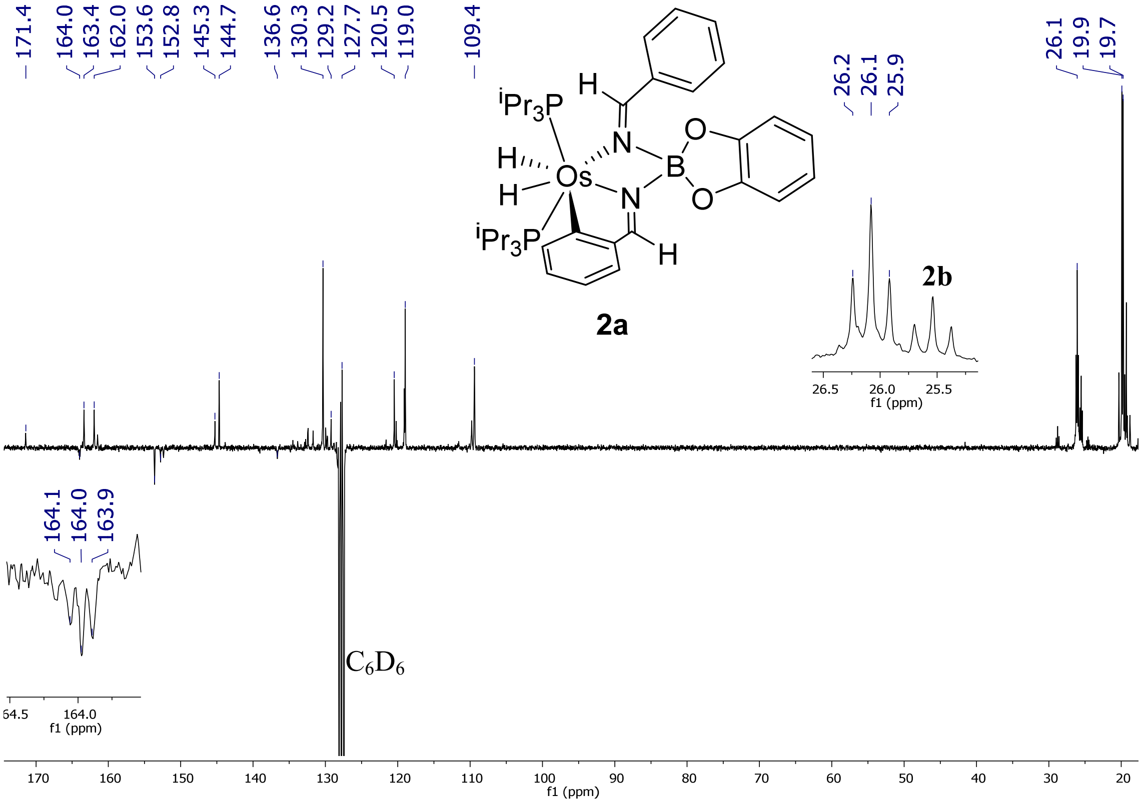

Figure S4. $^{13}C\{^{1}H\}$ APT NMR (75.48 MHz, C_6D_6, 298 K) spectrum of complex **2a**.

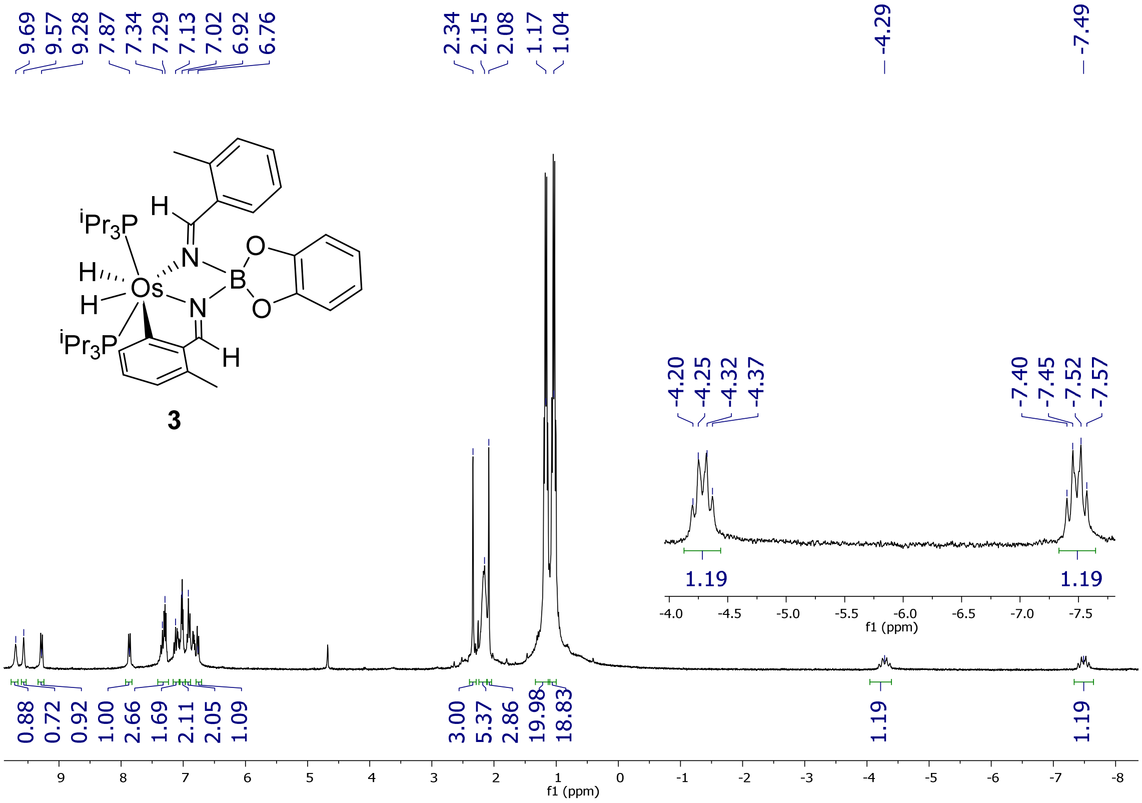

Figure S5. ^{1}H NMR (300.13 MHz, C_6D_6, 298 K) spectrum for complex **3**.

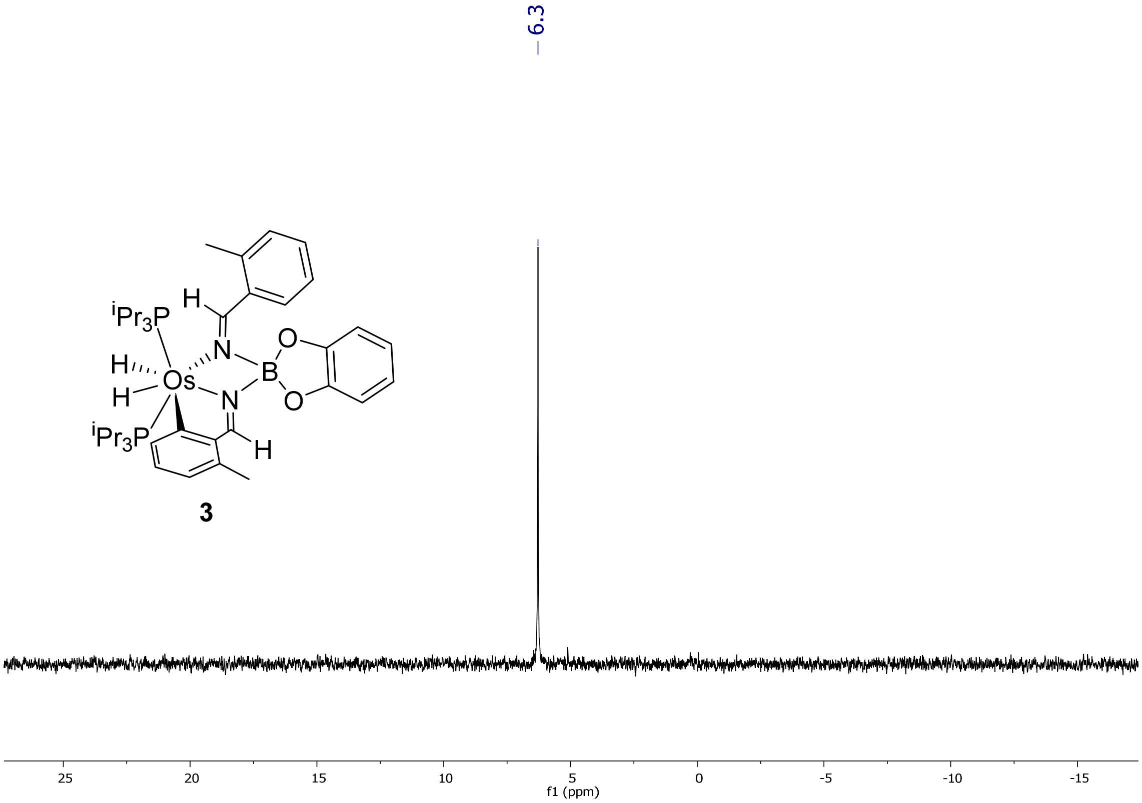

Figure S6. $^{31}P\{^{1}H\}$ NMR (121.50 MHz, C_6D_6, 298 K) spectrum for complex **3**.

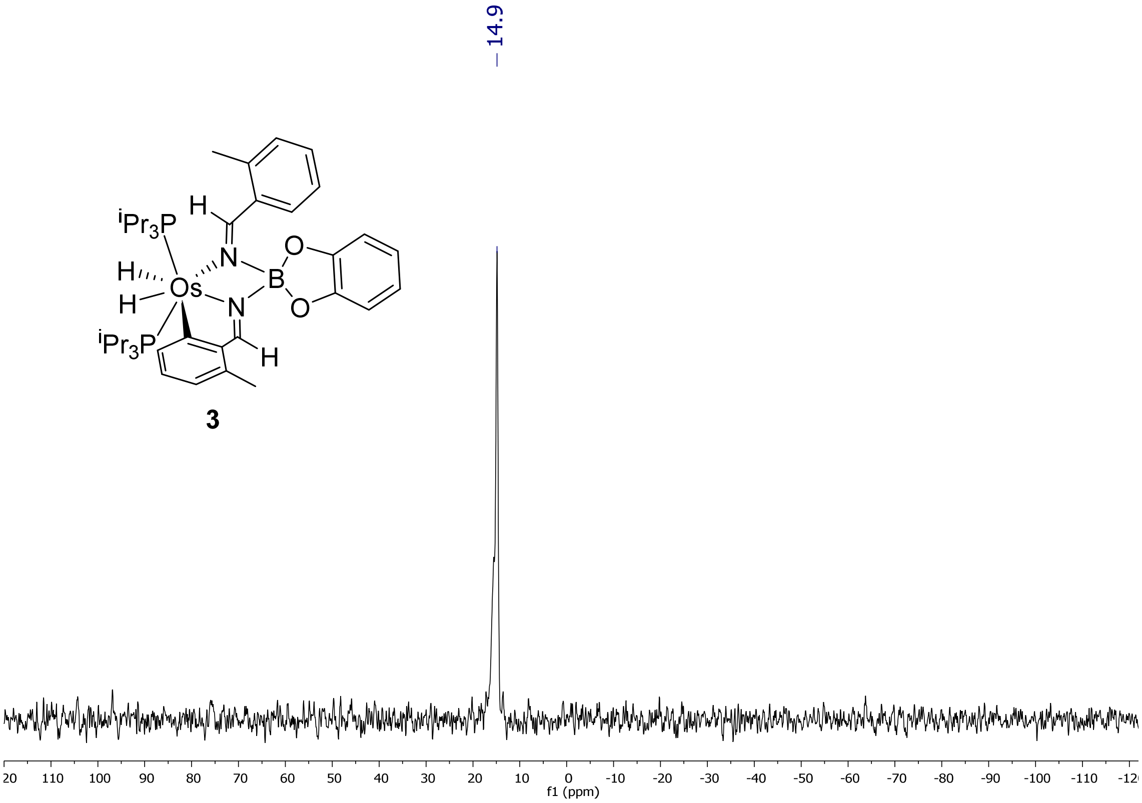

Figure S7. $^{11}B\{^{1}H\}$ APT NMR (96.29 MHz, C_6D_6, 298 K) spectrum of complex **3**.

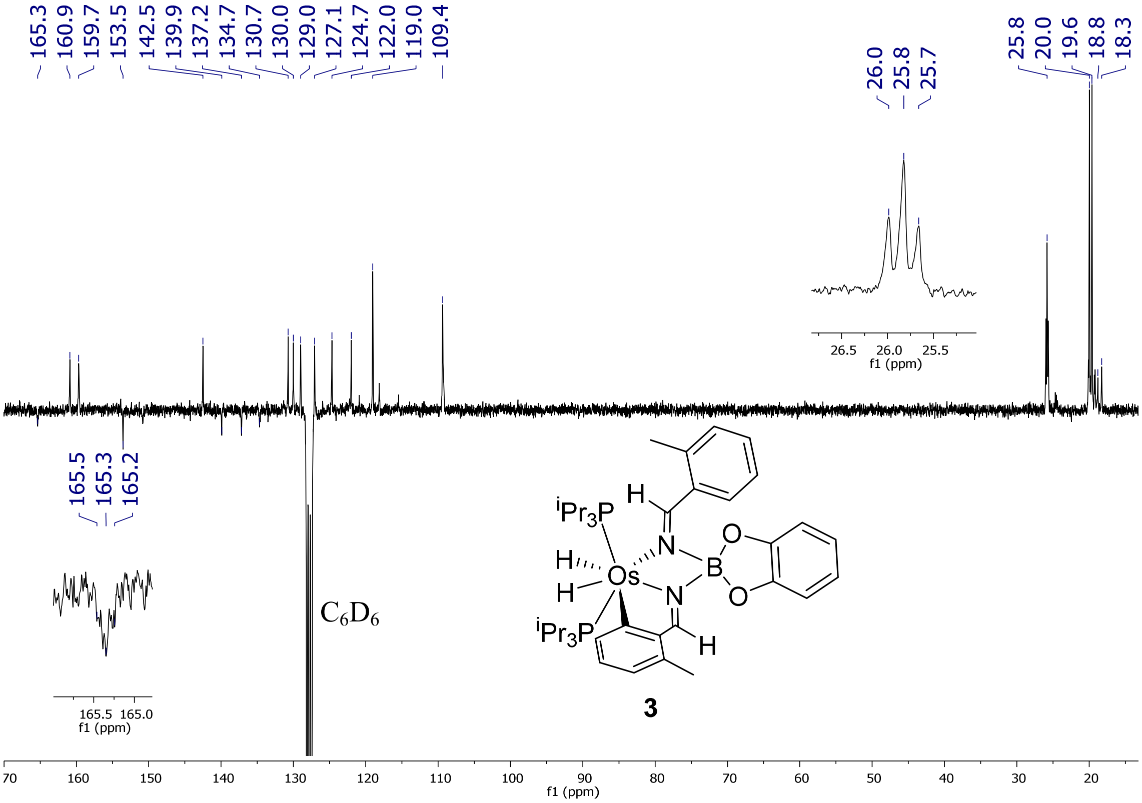

Figure S8. $^{13}C\{^{1}H\}$ APT NMR (75.48 MHz, C_6D_6, 298 K) spectrum of complex **3**.

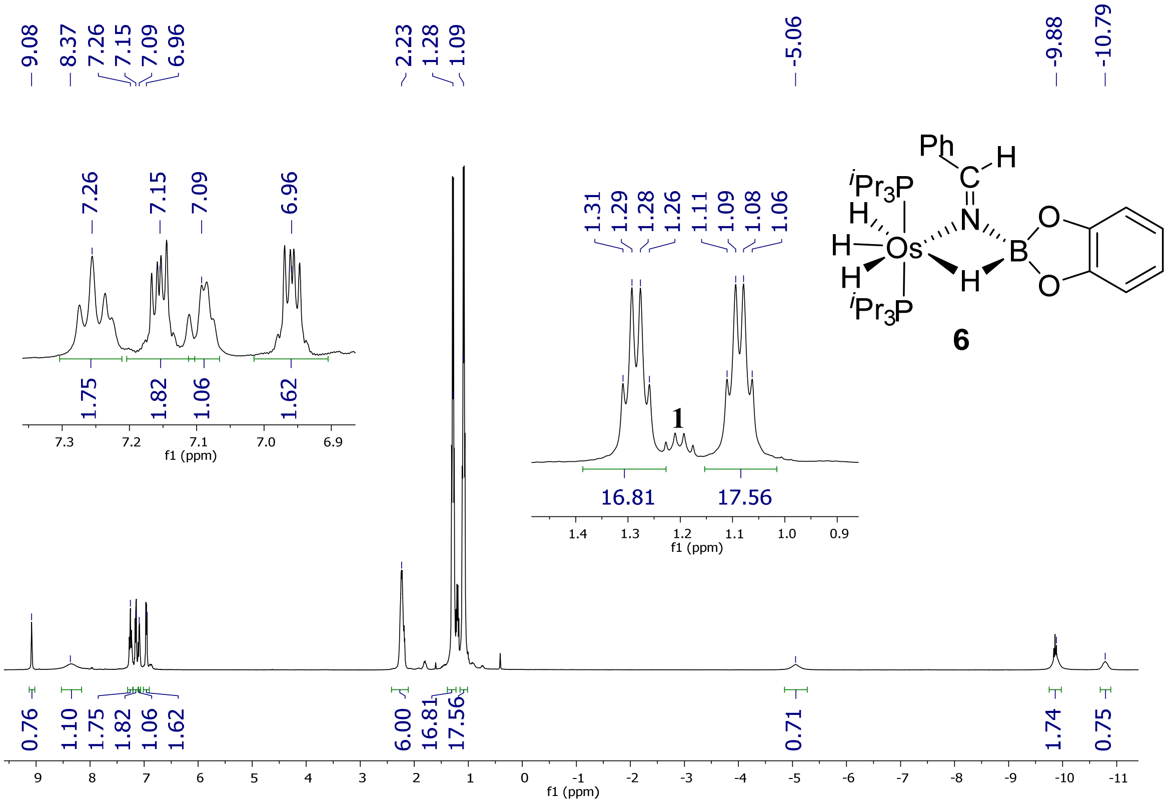

Figure S9. ^{1}H NMR (400.13 MHz, C_7D_8, 243 K) spectrum of complex **6**.

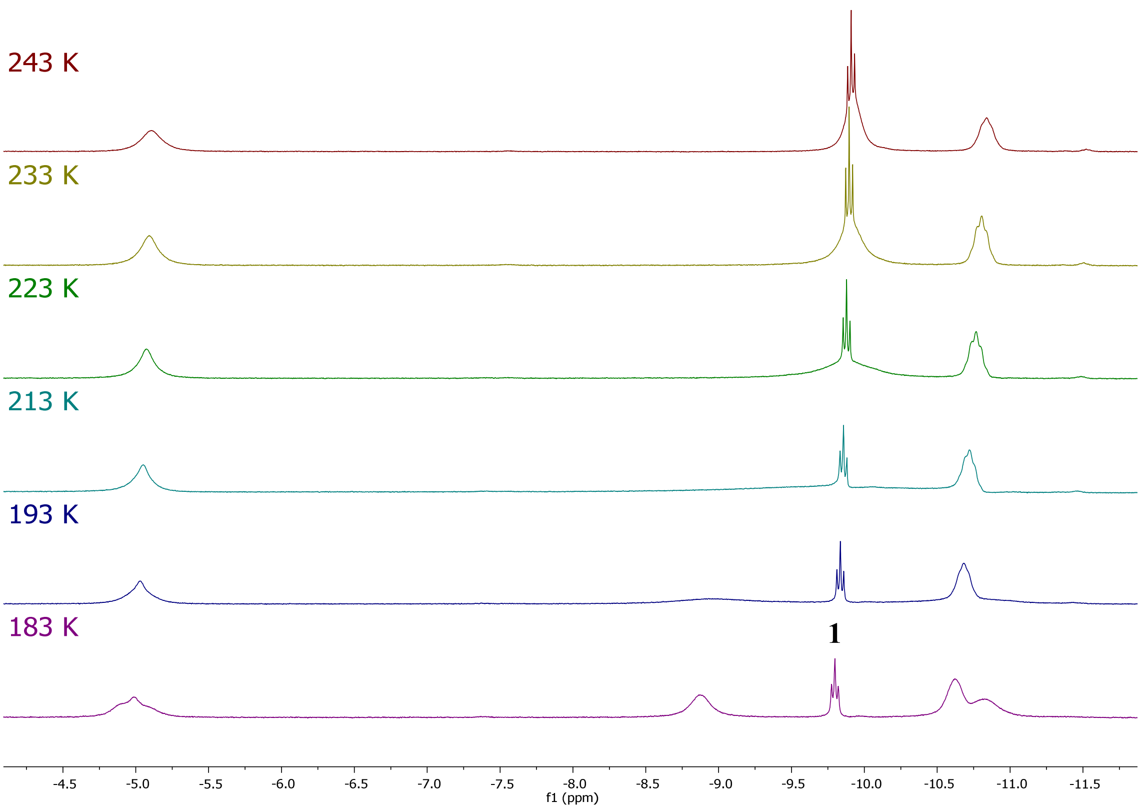

Figure S10. High-field region of the ^{1}H NMR (400.13 MHz, C_7D_8) spectrum of complex **6** between 243 and 183 K.

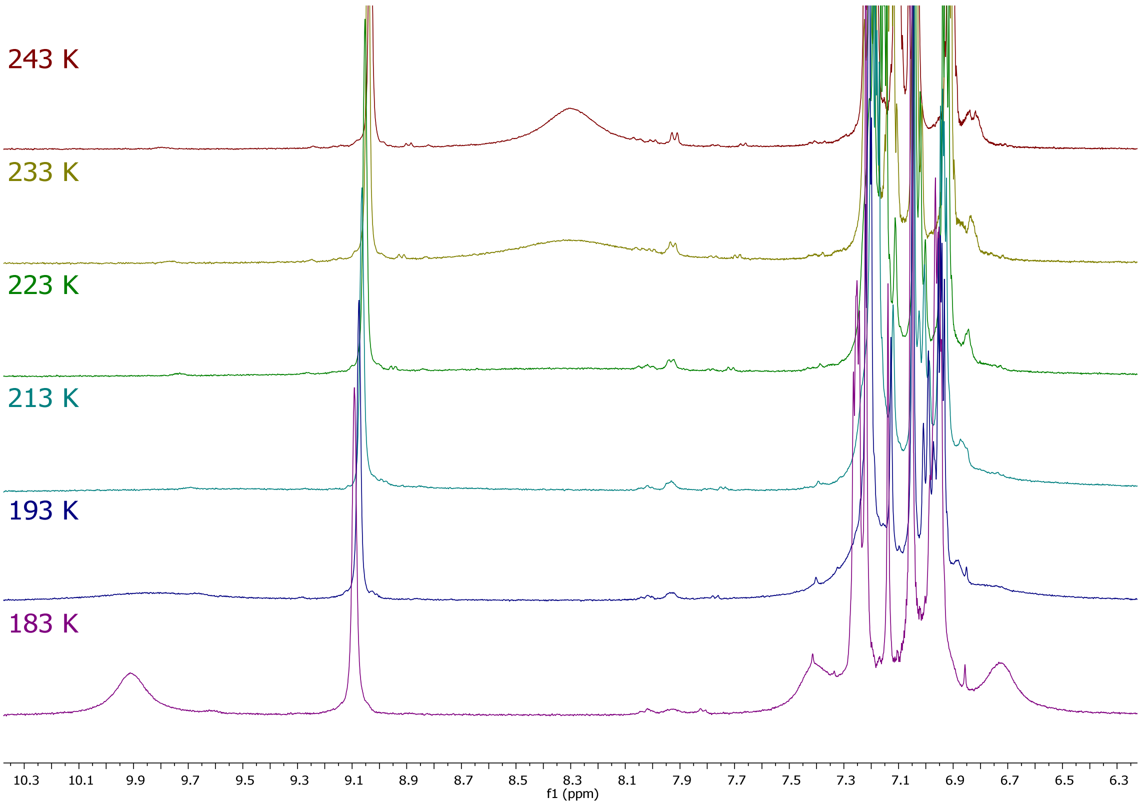

Figure S11. Low-field region of the ^{1}H NMR (400.13 MHz, C_7D_8) spectrum of complex **6** between 243 and 183 K.

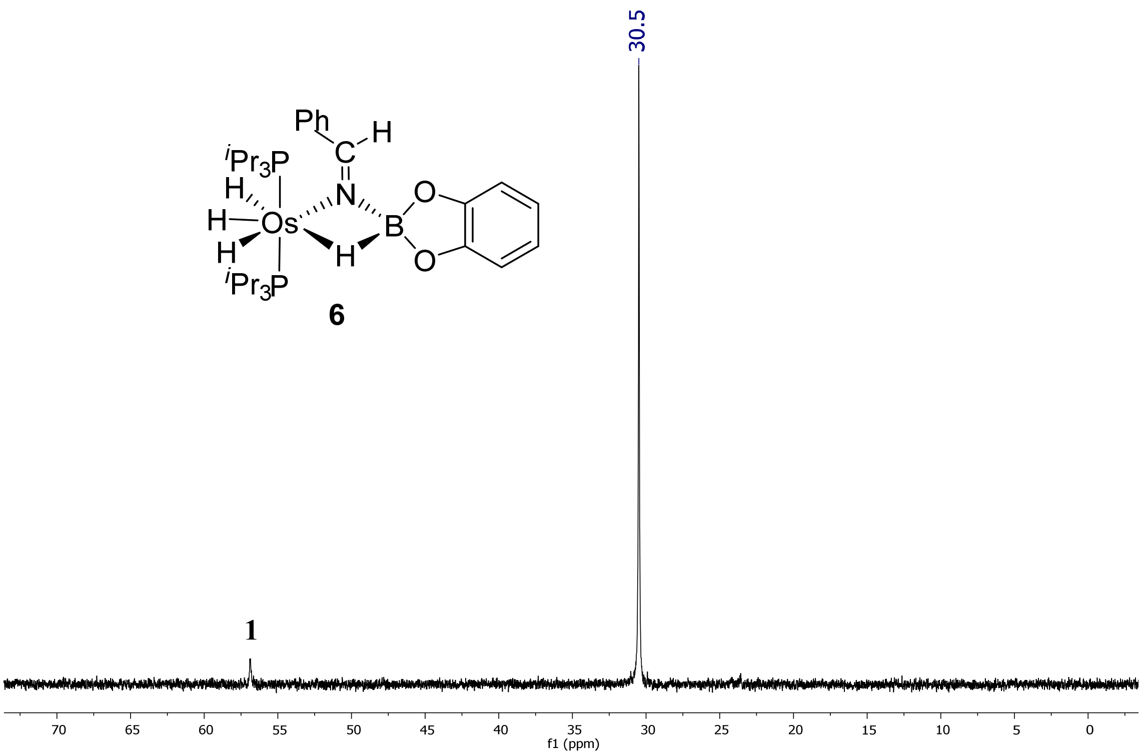

Figure S12. $^{31}P\{^{1}H\}$ NMR (161.98 MHz, C_7D_8, 243 K) spectrum for complex **6**.

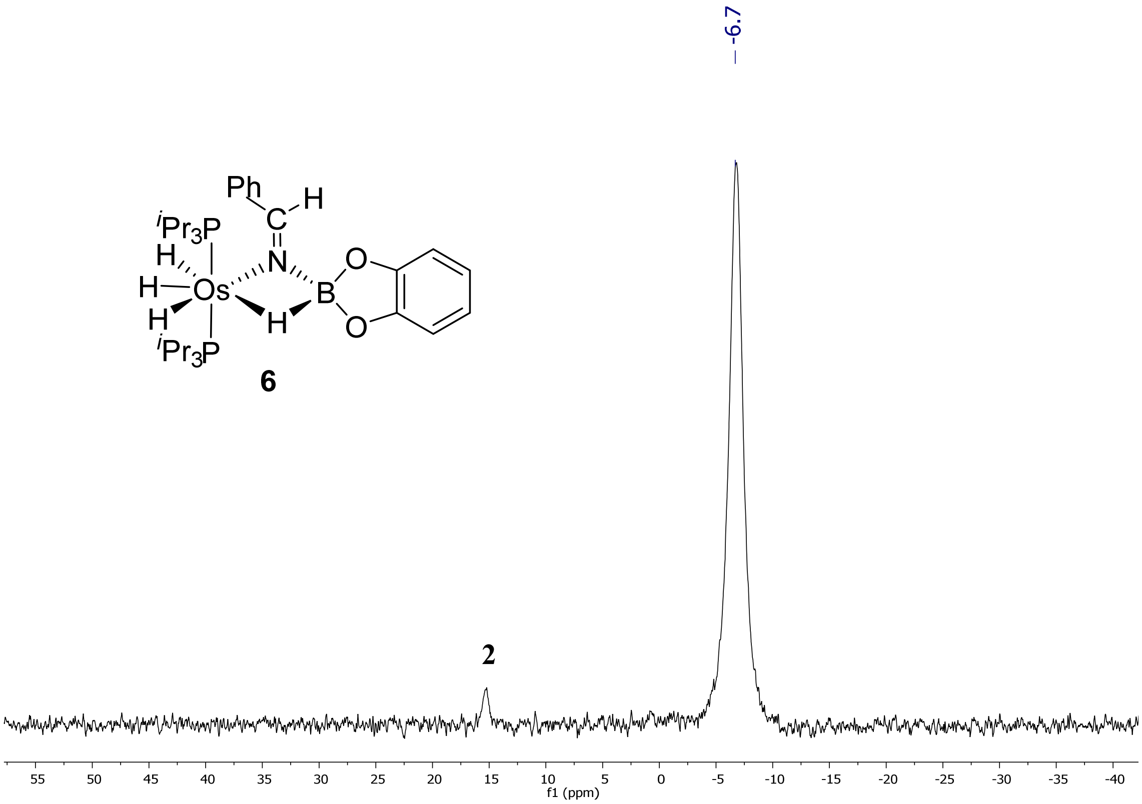

Figure S13. $^{11}B\{^{1}H\}$ NMR (128.38, MHz, C_7D_8, 243 K) spectrum for complex **6**.

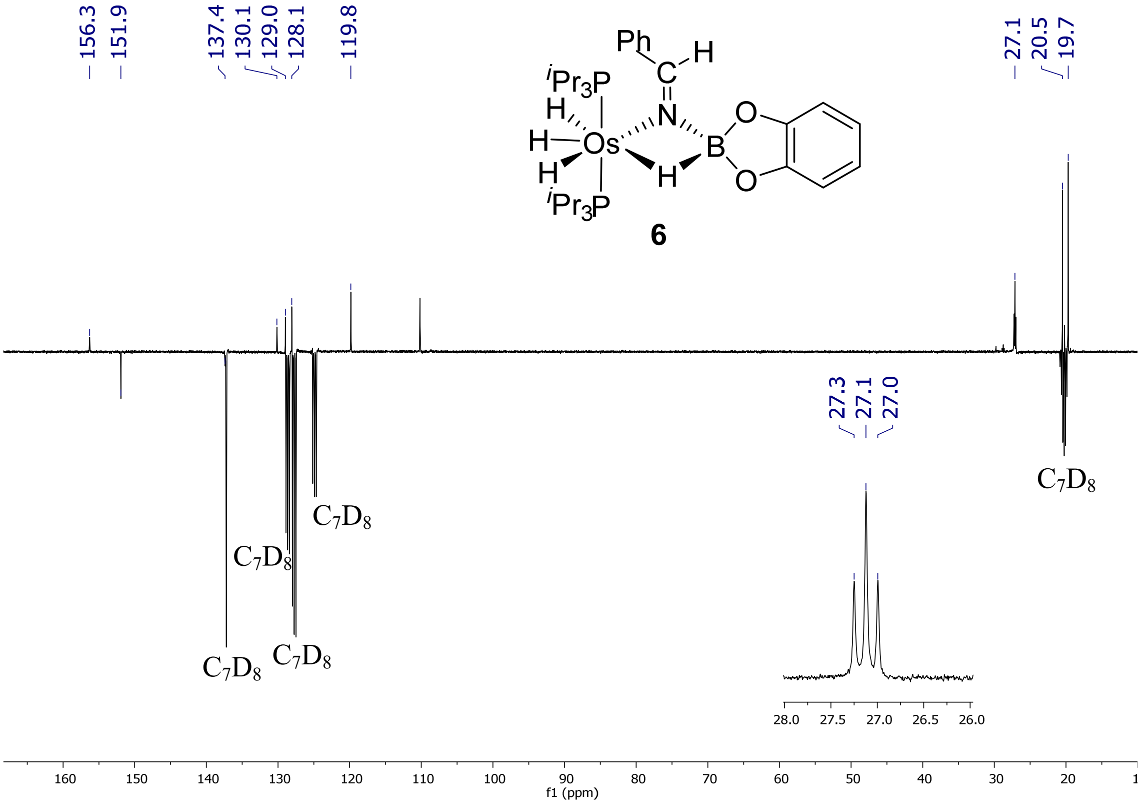

Figure S14. $^{13}C\{^1H\}$ APT NMR (100.62 MHz, C_7D_8, 243 K) spectrum for complex **6**.

Structural Analysis of Complex 2a. X-ray data were collected for the complex on a Bruker APEX DUO diffractometer equipped with a normal or fine focus, and 2.4 kW sealed tube source (Mo radiation, λ = 0.71073 Å). Data were collected over the complete sphere covering 0.3° in ω. Data were corrected for absorption by using a multiscan method applied with the SADABS program.[1] The structures were solved by Patterson or direct methods and refined by full-matrix least squares on F^2 with SHELXL2016,[2] including isotropic and subsequently anisotropic displacement parameters. The hydrogen atoms were observed in the last Fourier Maps or calculated and refined freely or using a restricted riding model. The hydride ligands were observed in the difference Fourier maps and refined with a restrained distance to osmium atoms. An isopropyl group was observed disordered and refined with two moieties, restrained geometry and complementary occupancy factors.

Crystal data for **2a**: $C_{38}H_{59}BN_2O_2OsP_2$, M_W 838.82, orange, irregular block (0.068 x 0.068 x 0.059 mm^3), triclinic, space group P-1, *a*: 12.401(3) Å, *b*: 15.419(3) Å, *c*: 20.211(4) Å, *α*: 88.602(3)°, *β*: 88.673(3)°, *γ*: 78.187(4)°, *V* = 3781.2(13) Å^3, *Z* = 4, *Z'* =

2, D_{calc}: 1.474 g cm^{-3}, F(000): 1712, T = 100(2) K, μ 3.491 mm^{-1}. 34229 measured reflections (2θ: 3-57°, ω scans 0.3°), 7 13445 unique (R_{int} = 0.1137); min./max. transm. Factors 0.637/0.862. Final agreement factors were R^1 = 0.0580 (7615 observed reflections, $I > 2\sigma(I)$) and wR^2 = 0.1409; data/restraints/parameters 13445/ 31/ 885; GoF = 0.962. Largest peak and hole 2.650 (close to osmium atoms) and -1.742 e/ $Å^3$.

Computational Details.

Geometries were fully optimized in solution using the Gaussian09[3] suite of programs at the B3LYP[4]/def2-SVP[5] level of theory using the D3 dispersion correction suggested by Grimme *et al.*[6] This level is denoted B3LYP-D3/def2-SVP. The PCM continuum model was used to model the effects of the solvent (toluene). Reactants, intermediates, and products were also characterized by frequency calculations and has positive definite Hessian matrices thus confirming that the computed structure is a minimum on the potential energy surface. Transition states were identified by having one imaginary frequency in the Hessian matrix. It was confirmed that transition states connect with the corresponding intermediates by means of application of an eigenvector corresponding to the imaginary frequency and subsequent optimization of the resulting structures. Gibbs energies were computed at 298.15 K and 1 atmosphere. All values collected in schemes and figures correspond to Gibbs energies in toluene in kcal mol^{-1}.

Wiberg Bond Indices (WBIs) have been computed using the natural bond orbital (NBO) method.[7] All AIM results described in this work correspond to calculations performed at the BP86-D3/6-31+G(d)/WTBS(for Os) level on the optimized geometry obtained at the BP86-D3/def2-SVP level. The WTBS (well-tempered basis sets)[8] have been recommended for AIM calculations involving transition metals.[9] The topology of the electron density was conducted using the AIMAll program package.[10]

The interaction between the transition metal fragment and the pincer ligand in complex **2a** has been investigated with the EDA-NOCV method,[11] which combines the energy decomposition analysis (EDA)[12] with the natural orbitals for chemical valence (NOCV)[13] methods. Within this approach, the interaction energy can be decomposed into the following physically meaningful terms:

$$\Delta E_{int} = \Delta E_{elstat} + \Delta E_{Pauli} + \Delta E_{orb} + \Delta E_{disp}$$

The term ΔE_{elstat} corresponds to the classical electrostatic interaction between the unperturbed charge distributions of the deformed reactants and is usually attractive. The Pauli repulsion ΔE_{Pauli} comprises the destabilizing interactions between occupied orbitals and is responsible for any steric repulsion. The orbital interaction ΔE_{orb} accounts for charge transfer (interaction between occupied orbitals on one moiety with unoccupied orbitals on the other, including HOMO–LUMO interactions) and polarization (empty-occupied orbital mixing on one fragment due to the presence of another fragment). Finally, the ΔE_{disp} term takes into account the interactions which are due to dispersion forces.

The EDA-NOCV method makes it possible to further partition the total orbital interactions into pairwise contributions of the orbital interactions. Details of the method can be found in the literature.[14]

The EDA-NOCV calculations were carried out using the BP86-D3/def2-SVP optimized geometry with the program package ADF 2017.01[15] using the same functional (BP86-D3) in conjunction with a triple-ζ-quality basis set using uncontracted Slater-type orbitals (STOs) augmented by two sets of polarization function with a frozen-core approximation for the core electrons.[16] An auxiliary set of s, p, d, f, and g STOs were used to fit the molecular densities and to represent the Coulomb and exchange potentials accurately in each SCF cycle.[17] Scalar relativistic effects were incorporated by applying the zeroth-order regular approximation (ZORA).[18] This level of theory is denoted BP86-D3/TZ2P//BP86-D3/def2-SVP.

Table S1. EDA-NOCV Results (in kcal/mol) Computed at the ZORA-BP86-D3/TZ2P//BP86-D3/def2-SVP Level

Fragments	$[OsH_2(P^iPr_3)_2]^+$ + $[CNN]^-$
ΔE_{int}	–290.3
ΔE_{Pauli}	420.2
$\Delta E_{elstat}{}^{a}$	–389.4 (54.8%)
$\Delta E_{orb}{}^{a}$	–279.0 (39.3%)
$\Delta E_{disp}{}^{a}$	–42.2 (5.9%)

[a]The values within parentheses indicate the percentage to the total attractive interaction energy.

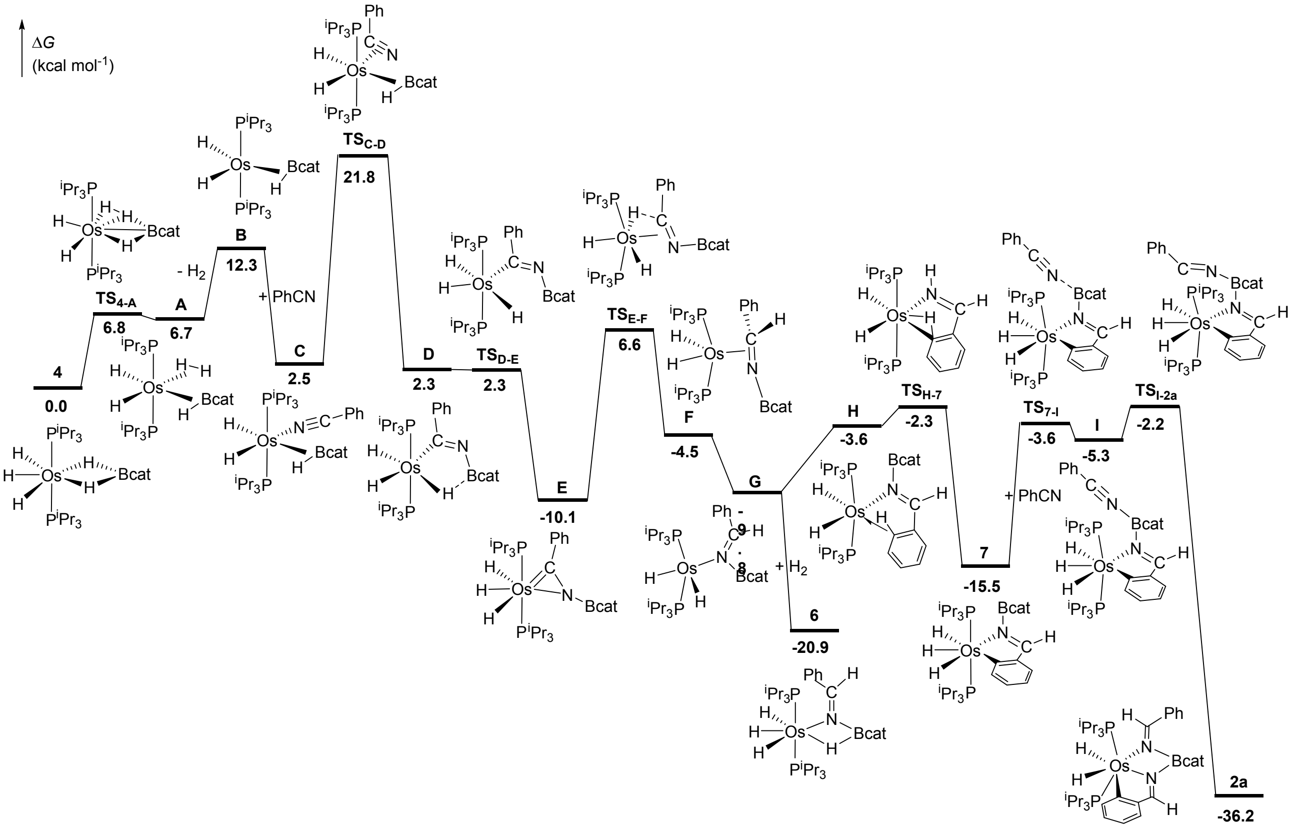

Figure S15. Computed energy profile for the formation of omplex **2a**

Energy values of the computed structures

Hydrogen

Zero-point correction= 0.009951 (Hartree/Particle)
Thermal correction to Energy= 0.012312
Thermal correction to Enthalpy= 0.013256
Thermal correction to Gibbs Free Energy= -0.001581
Sum of electronic and zero-point Energies= -1.163918
Sum of electronic and thermal Energies= -1.161557
Sum of electronic and thermal Enthalpies= -1.160613
Sum of electronic and thermal Free Energies= -1.175451

NCPh

Zero-point correction= 0.099366 (Hartree/Particle)
Thermal correction to Energy= 0.105432
Thermal correction to Enthalpy= 0.106376
Thermal correction to Gibbs Free Energy= 0.069106
Sum of electronic and zero-point Energies= -324.167387
Sum of electronic and thermal Energies= -324.161321
Sum of electronic and thermal Enthalpies= -324.160377
Sum of electronic and thermal Free Energies= -324.197647

4

Zero-point correction= 0.707838 (Hartree/Particle)
Thermal correction to Energy= 0.748023
Thermal correction to Enthalpy= 0.748967
Thermal correction to Gibbs Free Energy= 0.637060
Sum of electronic and zero-point Energies= -1892.586759
Sum of electronic and thermal Energies= -1892.546574
Sum of electronic and thermal Enthalpies= -1892.545629
Sum of electronic and thermal Free Energies= -1892.657537

TS_{4-A}

Zero-point correction= 0.703758 (Hartree/Particle)
Thermal correction to Energy= 0.743976
Thermal correction to Enthalpy= 0.744921
Thermal correction to Gibbs Free Energy= 0.631421
Sum of electronic and zero-point Energies= -1892.575599
Sum of electronic and thermal Energies= -1892.535381
Sum of electronic and thermal Enthalpies= -1892.534437
Sum of electronic and thermal Free Energies= -1892.647936

A

Zero-point correction= 0.705430 (Hartree/Particle)
Thermal correction to Energy= 0.746202
Thermal correction to Enthalpy= 0.747147
Thermal correction to Gibbs Free Energy= 0.633304
Sum of electronic and zero-point Energies= -1892.574752
Sum of electronic and thermal Energies= -1892.533980
Sum of electronic and thermal Enthalpies= -1892.533036
Sum of electronic and thermal Free Energies= -1892.646879

B

Zero-point correction= 0.689896 (Hartree/Particle)
Thermal correction to Energy= 0.729248
Thermal correction to Enthalpy= 0.730192
Thermal correction to Gibbs Free Energy= 0.620423
Sum of electronic and zero-point Energies= -1891.392966
Sum of electronic and thermal Energies= -1891.353614
Sum of electronic and thermal Enthalpies= -1891.352670
Sum of electronic and thermal Free Energies= -1891.462439

C

Zero-point correction= 0.792394 (Hartree/Particle)
Thermal correction to Energy= 0.838850
Thermal correction to Enthalpy= 0.839794
Thermal correction to Gibbs Free Energy= 0.712745
Sum of electronic and zero-point Energies= -2215.596088
Sum of electronic and thermal Energies= -2215.549631

Sum of electronic and thermal Enthalpies= -2215.548687
Sum of electronic and thermal Free Energies= -2215.675736

TS_{C-D}

Zero-point correction= 0.791999 (Hartree/Particle)
Thermal correction to Energy= 0.838049
Thermal correction to Enthalpy= 0.838993
Thermal correction to Gibbs Free Energy= 0.714941
Sum of electronic and zero-point Energies= -2215.567873
Sum of electronic and thermal Energies= -2215.521823
Sum of electronic and thermal Enthalpies= -2215.520879
Sum of electronic and thermal Free Energies= -2215.644931

D

Zero-point correction= 0.792415 (Hartree/Particle)
Thermal correction to Energy= 0.838402
Thermal correction to Enthalpy= 0.839346
Thermal correction to Gibbs Free Energy= 0.715223
Sum of electronic and zero-point Energies= -2215.598921
Sum of electronic and thermal Energies= -2215.552934
Sum of electronic and thermal Enthalpies= -2215.551990
Sum of electronic and thermal Free Energies= -2215.676113

TS_{D-E}

Zero-point correction= 0.790718 (Hartree/Particle)
Thermal correction to Energy= 0.836496
Thermal correction to Enthalpy= 0.837440
Thermal correction to Gibbs Free Energy= 0.714050
Sum of electronic and zero-point Energies= -2215.600494
Sum of electronic and thermal Energies= -2215.554716
Sum of electronic and thermal Enthalpies= -2215.553772
Sum of electronic and thermal Free Energies= -2215.677162

E

Zero-point correction= 0.792428 (Hartree/Particle)
Thermal correction to Energy= 0.838457
Thermal correction to Enthalpy= 0.839401
Thermal correction to Gibbs Free Energy= 0.713764
Sum of electronic and zero-point Energies= -2215.617209
Sum of electronic and thermal Energies= -2215.571181
Sum of electronic and thermal Enthalpies= -2215.570237
Sum of electronic and thermal Free Energies= -2215.695874

TS_{E-F}

Zero-point correction= 0.790461 (Hartree/Particle)
Thermal correction to Energy= 0.836508
Thermal correction to Enthalpy= 0.837453

Thermal correction to Gibbs Free Energy= 0.710453
Sum of electronic and zero-point Energies= -2215.589259
Sum of electronic and thermal Energies= -2215.543211
Sum of electronic and thermal Enthalpies= -2215.542267
Sum of electronic and thermal Free Energies= -2215.669267

F

Zero-point correction= 0.796044 (Hartree/Particle)
Thermal correction to Energy= 0.841798
Thermal correction to Enthalpy= 0.842742
Thermal correction to Gibbs Free Energy= 0.718757
Sum of electronic and zero-point Energies= -2215.609628
Sum of electronic and thermal Energies= -2215.563874
Sum of electronic and thermal Enthalpies= -2215.562930
Sum of electronic and thermal Free Energies= -2215.686916

G

Zero-point correction= 0.796506 (Hartree/Particle)
Thermal correction to Energy= 0.842337
Thermal correction to Enthalpy= 0.843281
Thermal correction to Gibbs Free Energy= 0.719870
Sum of electronic and zero-point Energies= -2215.618768
Sum of electronic and thermal Energies= -2215.572938
Sum of electronic and thermal Enthalpies= -2215.571993
Sum of electronic and thermal Free Energies= -2215.695404

6

Zero-point correction= 0.814391 (Hartree/Particle)
Thermal correction to Energy= 0.860948
Thermal correction to Enthalpy= 0.861892
Thermal correction to Gibbs Free Energy= 0.736127
Sum of electronic and zero-point Energies= -2216.810230
Sum of electronic and thermal Energies= -2216.763673
Sum of electronic and thermal Enthalpies= -2216.762729
Sum of electronic and thermal Free Energies= -2216.888494

H

Zero-point correction= 0.795181 (Hartree/Particle)
Thermal correction to Energy= 0.840525
Thermal correction to Enthalpy= 0.841469
Thermal correction to Gibbs Free Energy= 0.719164
Sum of electronic and zero-point Energies= -2215.609391
Sum of electronic and thermal Energies= -2215.564047
Sum of electronic and thermal Enthalpies= -2215.563103
Sum of electronic and thermal Free Energies= -2215.685408

TS_{H-7}

Zero-point correction= 0.791686 (Hartree/Particle)
Thermal correction to Energy= 0.836786
Thermal correction to Enthalpy= 0.837731
Thermal correction to Gibbs Free Energy= 0.716105
Sum of electronic and zero-point Energies= -2215.607784
Sum of electronic and thermal Energies= -2215.562684
Sum of electronic and thermal Enthalpies= -2215.561740
Sum of electronic and thermal Free Energies= -2215.683365

7

Zero-point correction= 0.793977 (Hartree/Particle)
Thermal correction to Energy= 0.839397
Thermal correction to Enthalpy= 0.840342
Thermal correction to Gibbs Free Energy= 0.717821
Sum of electronic and zero-point Energies= -2215.628285
Sum of electronic and thermal Energies= -2215.582865
Sum of electronic and thermal Enthalpies= -2215.581920
Sum of electronic and thermal Free Energies= -2215.704441

TS_{7-I}

Zero-point correction= 0.894853 (Hartree/Particle)
Thermal correction to Energy= 0.947015
Thermal correction to Enthalpy= 0.947959
Thermal correction to Gibbs Free Energy= 0.810378
Sum of electronic and zero-point Energies= -2539.798609
Sum of electronic and thermal Energies= -2539.746448
Sum of electronic and thermal Enthalpies= -2539.745503
Sum of electronic and thermal Free Energies= -2539.883085

I

Zero-point correction= 0.895240 (Hartree/Particle)
Thermal correction to Energy= 0.947945
Thermal correction to Enthalpy= 0.948889
Thermal correction to Gibbs Free Energy= 0.809538
Sum of electronic and zero-point Energies= -2539.800173
Sum of electronic and thermal Energies= -2539.747468
Sum of electronic and thermal Enthalpies= -2539.746524
Sum of electronic and thermal Free Energies= -2539.885876

TS_{I-2a}

Zero-point correction= 0.894717 (Hartree/Particle)
Thermal correction to Energy= 0.946542
Thermal correction to Enthalpy= 0.947486
Thermal correction to Gibbs Free Energy= 0.811294
Sum of electronic and zero-point Energies= -2539.797387
Sum of electronic and thermal Energies= -2539.745563

Sum of electronic and thermal Enthalpies= -2539.744618
Sum of electronic and thermal Free Energies= -2539.880810

2a

Zero-point correction= 0.900885 (Hartree/Particle)
Thermal correction to Energy= 0.952254
Thermal correction to Enthalpy= 0.953198
Thermal correction to Gibbs Free Energy= 0.818303
Sum of electronic and zero-point Energies= -2539.852415
Sum of electronic and thermal Energies= -2539.801047
Sum of electronic and thermal Enthalpies= -2539.800102
Sum of electronic and thermal Free Energies= -2539.934997

References

(1) Blessing, R. H. *Acta Crystallogr.* **1995**, *A51*, 33. SADABS: Area-detector absorption correction; Bruker-AXS, Madison, WI, 1996.

(2) SHELXL-2016/6. Sheldrick, G. M. *Acta Cryst.* **2008**, *A64*, 112-122.

(3) Gaussian 09, Revision D.01, Frisch, M. J.; Trucks, G. W.; Schlegel, H. B.; Scuseria, G. E.; Robb, M. A.; Cheeseman, J. R.; Scalmani, G.; Barone, V.; Mennucci, B.; Petersson, G. A.; Nakatsuji, H.; Caricato, M.; Li, X.; Hratchian, H. P.; Izmaylov, A. F.; Bloino, J.; Zheng, G.; Sonnenberg, J. L.; Hada, M.; Ehara, M.; Toyota, K.; Fukuda, R.; Hasegawa, J.; Ishida, M.; Nakajima, T.; Honda, Y.; Kitao, O.; Nakai, H.; Vreven, T.; Montgomery, J. A., Jr.; Peralta, J. E.; Ogliaro, F.; Bearpark, M.; Heyd, J. J.; Brothers, E.; Kudin, K. N.; Staroverov, V. N.; Kobayashi, R.; Normand, J.; Raghavachari, K.; Rendell, A.; Burant, J. C.; Iyengar, S. S.; Tomasi, J.; Cossi, M.; Rega, N.; Millam, J. M.; Klene, M.; Knox, J. E.; Cross, J. B.; Bakken, V.; Adamo, C.; Jaramillo, J.; Gomperts, R.; Stratmann, R. E.; Yazyev, O.; Austin, A. J.; Cammi, R.; Pomelli, C.; Ochterski, J. W.; Martin, R. L.; Morokuma, K.; Zakrzewski, V. G.; Voth, G. A.; Salvador, P.; Dannenberg, J. J.; Dapprich, S.; Daniels, A. D.; Farkas, Ö.; Foresman, J. B.; Ortiz, J. V.; Cioslowski, J.; Fox, D. J. Gaussian, Inc., Wallingford CT, 2009.

(4) (a) Becke, *A. D.* Density-functional exchange-energy approximation with correct asymptotic behavior. *Phys. Rev. A* **1988**, *38*, 3098-3100. (b) Perdew, J. P. Density-functional approximation for the correlation energy of the inhomogeneous electron gas. *Phys. Rev. B* **1986**, *33*, 8822-8824.

(5) Weigend, F.; Ahlrichs, R. Balanced basis sets of split valence, triple zeta valence and quadruple zeta valence quality for H to Rn: Design and assessment of accuracy. *Phys. Chem. Chem. Phys*. **2005**, *7*, 3297-3305.

(6) Grimme, S.; Antony, J.; Ehrlich, S.; Krieg, H. A consistent and accurate ab initio parametrization of density functional dispersion correction (DFT-D) for the 94 elements H-Pu. *J. Chem. Phys*. **2010**, *132*, 154104.

(7) (a) Foster, J. P.; Weinhold, F. Natural Hybrid Orbitals. *J. Am. Chem. Soc.* **1980**, *102*, 7211–7218. (b) Reed, A. E.; Weinhold, F. Natural Localized Molecular Orbitals. *J. Chem. Phys.* **1985**, *83*, 1736–1740. (c) Reed, A. E.; Weinstock, R. B.; Weinhold, F. Natural Population Analysis. *J. Chem. Phys.* **1985**, *83*, 735–746. (d) Reed, A. E.; Curtiss, L. A.; Weinhold, F. Intermolecular Interactions from a Natural Bond Orbital, Donor-Acceptor Viewpoint. *Chem. Rev.* **1988**, *88*, 899–926.

(8) (a) Huzinaga, S.; Miguel, B. A comparison of the geometrical sequence formula and the well-tempered formulas for generating GTO basis orbital exponents. *Chem. Phys. Lett.* **1990**, *175*, 289-291. (b) Huzinaga, S.; Klobukowski, M. Well-Tempered Gaussian Basis Sets for the Calculation of Matrix Hartree-Fock Wavefunctions. *Chem. Phys. Lett.* **1993**, *212*, 260–264.

(9) Cabeza, J. A.; Van der Maelen, J. F.; García-Granda, S. Topological Analysis of the Electron Density in the N-Heterocyclic Carbene Triruthenium Cluster [$Ru_3(\mu\text{-}H)_2(\mu_3$-MeImCH)$(CO)_9$] ($Me_2Im$ = 1,3-dimethylimidazol-2-ylidene). *Organometallics* **2009**, *28*, 3666–3672 and references therein.

(10) Keith, T. A. AIMAll, 2010, http://tkgristmill.com.

(11) Mitoraj, M. P.; Michalak, A.; Ziegler, T. A Combined Charge and Energy Decomposition Scheme for Bond Analysis. *J. Chem. Theory Comput.* **2009**, *5*, 962-975.

(12) For a recent review, see: von Hopffgarten, M.; Frenking, G. Energy decomposition analysis. *WIREs Comput. Mol. Sci.* **2012**, *2*, 43-62.

(13) Mitoraj, M. P.; Michalak, A. Natural orbitals for chemical valence as descriptors of chemical bonding in transition metal complexes. *J. Mol. Model.* **2007**, *13*, 347-355.

(14) See, for instance: (a) Mitoraj, M. P.; Michalak, A.; Ziegler, T. On the Nature of the Agostic Bond between Metal Centers and β-Hydrogen Atoms in Alkyl Complexes. An Analysis Based on the Extended Transition State Method and the Natural Orbitals for Chemical Valence Scheme (ETS-NOCV). *Organometallics* **2009**, *28*, 3727-3733. (b) Thi, A. N. N.; Frenking, G. Transition-Metal Complexes of Tetrylones [$(CO)_5W\text{-}E(PPh_3)_2$] and Tetrylenes [$(CO)_5W$-NHE] (E = C–Pb): A Theoretical Study. *Chem. Eur. J.* **2012**, *18*, 12733-12748. (c) Parafiniuk, M.; Mitoraj, M. P. Origin of Binding of Ammonia–Borane to Transition-Metal-Based Catalysts: An Insight from the Charge and Energy Decomposition Method ETS-NOCV. *Organometallics* **2013**, *32*, 4103-4113.

(15) ADF program: www.scm.com.

(16) Snijders, J. G.; Vernooijs, P.; Baerends, E. J. Roothaan-Hartree-Fock-Slater Atomic Wave Functions: Single-Zeta, Double-Zeta, and Extended Slater-Type Basis Sets for $_{87}Fr$-$_{103}Lr$. *At. Data. Nucl. Data Tables* **1982**, *26*, 483-509.

(17) Krijn, A.; Baerends, E. J.; Fit Functions in the HFS-Method, Internal Report (in Dutch), Vrije Universiteit Amsterdam, The Netherlands, **1984**.

(18) (a) van Lenthe, E.; Baerends, E. J.; Snijders, J. G. Relativistic regular two-component Hamiltonians. *J. Chem. Phys.* **1993**, *99*, 4597-4610. (b) van Lenthe, E.; Baerends, E. J.; Snijders, J. G. Relativistic total energy using regular approximations. *J. Chem. Phys.* **1994**, *101*, 9783-9792. (c) van Lenthe, E.; Ehlers, A.; Baerends, E. J. Geometry optimizations in the zero order regular approximation for relativistic effects. *J. Chem. Phys.* **1999**, *110*, 8943-8953.
